# Carbon nano-dot for cancer studies as dual nano-sensor for imaging intracellular temperature or pH variation

**DOI:** 10.1038/s41598-021-03686-x

**Published:** 2021-12-21

**Authors:** Trilochan Gadly, Goutam Chakraborty, Mrityunjay Tyagi, Birija S. Patro, Bijaideep Dutta, Akhilesh Potnis, Pallavi Chandwadkar, Celin Acharya, Shishu Kant Suman, Archana Mukherjee, Suman Neogy, Amey Wadawale, Srikant Sahoo, Nitish Chauhan, Sunil K. Ghosh

**Affiliations:** 1 Bio-Organic Division, Bhabha Atomic Research Centre, Trombay, Mumbai, 400085 India; 2 Laser and Plasma Technology Division, Bhabha Atomic Research Centre, Trombay, Mumbai, 400085 India; 3 Chemistry Division, Bhabha Atomic Research Centre, Trombay, Mumbai, 400085 India; 4 Molecular Biology Division, Bhabha Atomic Research Centre, Trombay, Mumbai, 400085 India; 5 Radiopharmaceuticals Division, Bhabha Atomic Research Centre, Trombay, Mumbai, 400085 India; 6 Material Science Division, Bhabha Atomic Research Centre, Trombay, Mumbai, 400085 India; 7 Analytical Chemistry Division, Bhabha Atomic Research Centre, Trombay, Mumbai, 400085 India

**Keywords:** Cell biology, Nanoscience and technology

## Abstract

Cellular temperature and pH govern many cellular physiologies, especially of cancer cells. Besides, attaining higher cellular temperature plays key role in therapeutic efficacy of hyperthermia treatment of cancer. This requires bio-compatible, non-toxic and sensitive probe with dual sensing ability to detect temperature and pH variations. In this regard, fluorescence based nano-sensors for cancer studies play an important role. Therefore, a facile green synthesis of orange carbon nano-dots (CND) with high quantum yield of 90% was achieved and its application as dual nano-sensor for imaging intracellular temperature and pH was explored. CND was synthesized from readily available, bio-compatible citric acid and rhodamine 6G hydrazide using solvent-free and simple heating technique requiring purification by dialysis. Although the particle size of 19 nm (which is quite large for CND) was observed yet CND exhibits no surface defects leading to decrease in photoluminescence (PL). On the contrary, very high fluorescence was observed along with good photo-stability. Temperature and pH dependent fluorescence studies show linearity in fluorescence intensity which was replicated in breast cancer cells. In addition, molecular nature of PL of CND was established using pH dependent fluorescence study. Together, the current investigation showed synthesis of highly fluorescent orange CND, which acts as a sensitive bio-imaging probe: an optical nano-thermal or nano-pH sensor for cancer-related studies.

## Introduction

**Figure 1:**
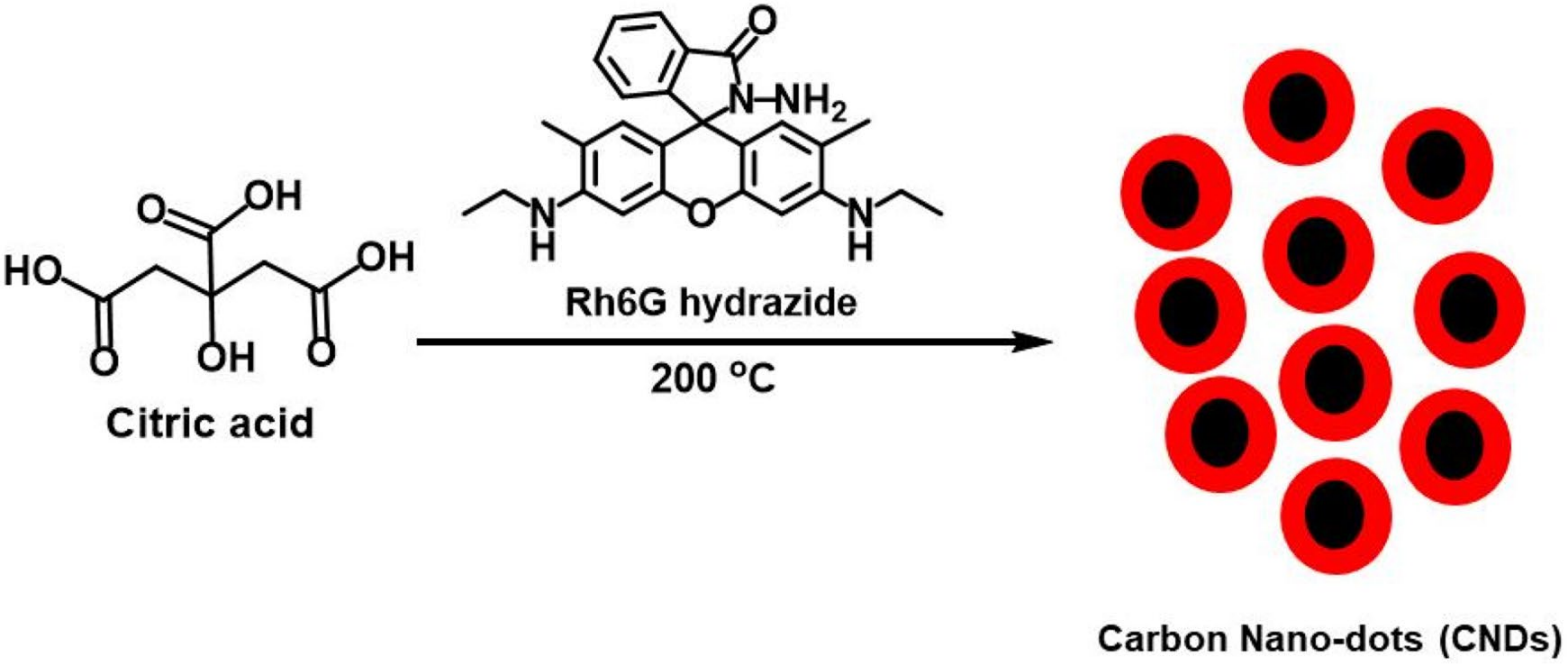
Synthesis of CND (ChemDraw Professional 16.0 was used to draw in this figure).

Variations in temperature and pH are markers of cellular events^1,2^. Intracellular pH and temperature values regulate pathological and physiological processes and disruptive changes of such parameters^3^ are indicators of functional disorder such as cancer^4^. Assessment of cellular pH and temperature are essential in cancer metastasis and hyperthermia treatment studies and this is possible if temperature and pH fluctuations in cancer cells are evaluated with sensitive probes such as non-invasive or non-destructive optical nano-sensors. Thus, fluorescent carbon nanodots (CNDs)^5^ are ideal nano-probes due to their bio-compatibility, non-toxicity, chemical and photo-stability which makes them suitable for various applications such as in-vivo animal bio-imaging, light emitting diodes, optical sensing and photo-catalysis^6–8^. The CNDs, which comprises mostly carbon, are typically less than 10 nm in diameter and can be produced by breaking down larger carbon structures (top-down approach) or by building from smaller carbon precursors (bottom-up approach).The latter process involves either combustion/thermal or supported synthesis/microwave methods/solvothermal process during which the CNDs are formed from molecular precursors. The primary challenge in the synthesis of CND by bottom-up processes is the size-control of products of variable polydispersity and properties^9^. The secondary challenge is attainment of high fluorescence of CND which is generally governed by quantum and physio-chemical processes^10^. In order to achieve conjugated sp^2^-domain conducive for orange and red emissions, the size of the particle needs to be increased. However, large sized particles usually contain huge surface defects, leading to non-efficient emissions^11^. Quantum yield (QY) exceeding 50% have seldom been reported for large CNDs in the orange-red regime and hence improvement for their development for widespread applications is warranted^12^. Therefore, rhodamine based fluorescence moieties appear to be particularly attractive due to their excellent spectroscopic properties, high fluorescence quantum yield and high photostability^13^. Generally, the spirolactam ring of rhodamine-based fluorescence probe is non-fluorescent and colorless in neutral or alkaline medium, whereas ring opening leads to strong fluorescence emission and pink coloration either on account of chelation to metal or at low pH or acidic conditions (Fig. 1)^14–16^. Previous studies show that optical performance of CNDs primarily depends on carbon core, surface states^17^ and molecular fluorophores^18^. Indeed, only weak fluorescence is observed from “naked” CND. It has been shown that citric acid can easily react with amines (especially primary amines) forming strongly luminescent molecular fluorophore, though mostly emitting in the blue region. Thus in our present study, to achieve remarkable enhancement of the fluorescence in CNDs, citric acid was covalently functionalized with rhodamine 6G (also known as rhodamine 590 or Rh6G) hydrazide. Since spirolactam ring of Rh6G hydrazide opens in acidic conditions hence, this moiety present in CND has the ability to sense pH changes. Moreover, fluorophores display molecular flexibility at elevated temperature which affects fluorescence intensity and therefore, temperature changes can also be detected using variations occurring in PL intensity. However, there are few reports of a bio-compatible, water soluble, non-toxic fluorescent nano-sensor which has the ability to detect either thermal or pH fluctuations inside cells^19^. Simultaneous detections of intracellular temperature and pH is a challenging task since intracellular pH is a varying parameter and photo-luminescent materials usually change their optical property such as emission intensity depending on environment. Thus luminescent sensors having high sensitivity towards temperature and low sensitivity towards pH are highly desirable. This can be achieved by synthesizing photo-luminescent materials having high quantum yield (QY) which can make temperature sensitivity more compared with pH sensitivity^20^. Hence Rh6G hydrazide and citric acid based CND fulfils the conditions required for a dual nano-sensor to detect intracellular temperature and/or pH for cancer studies owing to its high QY of 90%. In the past decades, technologies were developed extensively to non-invasively measure intra- and/or extracellular pH in cancer^21^. Quantitative imaging of distribution of acidity helps in understanding the role of tumor microenvironment in cancer progression and metastasis, which is associated with switching metabolism to glycolysis in cancer cells for generating acidic environment. The CND developed in the current investigation is helpful in measuring variation in pH in cancer cells under fixed temperature and also in measuring fluctuations in temperature in cancer cells at constant pH.

## Results

Rh6G hydrazide (CCDC 2051200, Fig. 1) was prepared^22,23^ from Rh6G dye by refluxing it with hydrazine hydrate for 6 h in methanol and characterized by ^1^H NMR (Fig. S1), ^13^C NMR (Fig. S2), single crystal XRD (Fig. S3, Tables S1–S3)^24–27^. and Fourier Transform Infra-Red spectrometer (FT-IR). CNDs were prepared from citric acid and Rh6G hydrazide by heating them at 200 °C in a heating mantle (Fig. 1).

**Figure 2:**
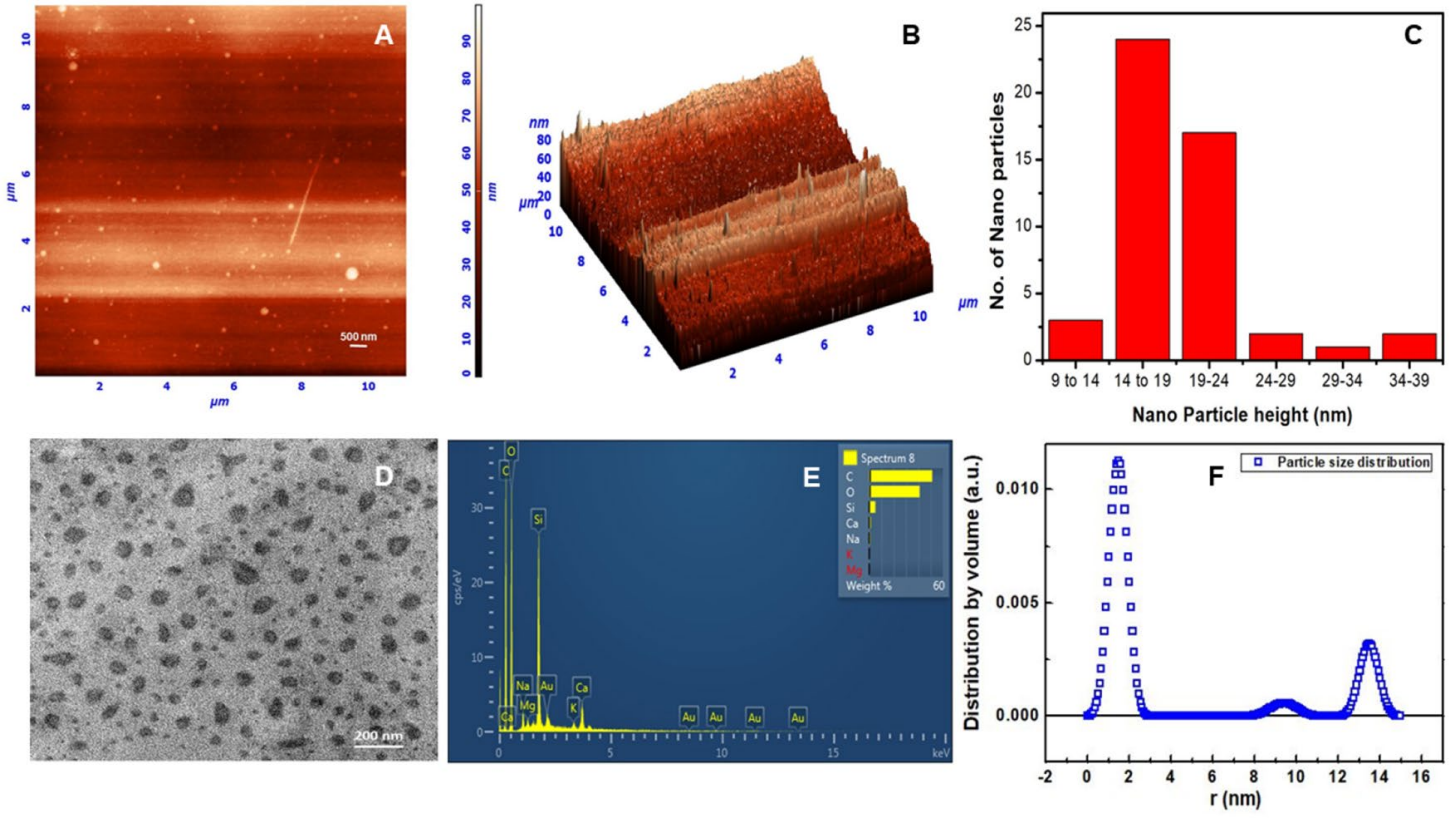
(**A**) AFM of CND (**B**) Three dimensional height projection of CND (**C**) Particle height distribution of CNDs from AFM (**D**) TEM image of CND E) EDX spectrum of CND (**F**) Particle size distribution weighted by volume of CNDs from small angle scattering (SAXS) data obtained by indirect fourier transform (IFT) software.

Dialysis of the desired CNDs gave high QY (0.90 or 90%) and as prepared dialysate without further purification was used for all subsequent experiments and analysis. The CNDs were characterized using ^1^H NMR (Fig. S4), ^13^C NMR (Fig. S5) and FT-IR (Fig. S6 and Table S4), elemental analysis (Table S5) and Energy Dispersive X-ray Analysis (EDX) spectroscopy (Figs. 2E, S7). The particle size distribution^28^, morphology and charge of the CNDs were established using Atomic Force Microscopy (AFM) (Figs. 2A–C, S8), Transmission Electron Microscopy (TEM) (Figs. 2D, S9), Scanning Electron Microscopy (SEM) (Fig. S10) and zeta potential analysis (Fig. S11). The particle size distribution of CND using AFM and TEM studies confirmed the prevalence of particle size heterogeneity. The electron diffraction pattern for crystalline structure was not observed in TEM inferring that CND is amorphous in nature (Fig. S9A). Wang et al. has reported synthesis of large sized CND (15–20 nm diameter) from sonication of glucose under alkaline conditions. The amorphous structure was ascertained from the absence of long-range pattern of atomic positions^29^. Indeed, the CND was found to lack crystalline structure in TEM owing to its amorphous nature. CNDs reported in the literature have hinted at the presence of graphitic structures forming core and surface states as shell structure^30^. TEM studies of our CND further elucidates the absence of core–shell type structure from the uniformity of contrast in CND images (Fig. S9B). During TEM viewing, however, agglomeration and self-assembly pattern (Fig. S9B,C) was also observed which led to conclusion that there may be some crystalline structure present in CND.

### .

Further characterization of the synthesized CND using small angle X-ray scattering (SAXS) and wide angle X-ray scattering experiments (Fig. S12) revealed the presence of both amorphous and crystalline forms. Amorphous nature of CND is clearly indicated from the SAXS study (Fig. S12A) and due to the presence of broad peaks between 5° and 35° in WAXS study (Fig. S12B). However sharp peaks at 18.6°, 19.9° and 21.4° also point towards some crystalline structure present in CND (Fig. S12B)^31^. The zeta potential of CND in ethanol was + 1.2 mV indicating positive surface potential of CND. The development of positive charge can be attributed to protonation of the amide and/or amine groups generated from Rh6G hydrazide. However, this result is in sharp contrast with the one obtained by Qu et al.^11^ where the zeta potentials of the orange CND were found to be − 17.3 and − 35.7 mV respectively. This further implies the absence of negative carboxylate groups resulting due to amide linkages between citric acid and Rh6G hydrazide. The stability of any colloidal system can be attributed to any of the two factors namely steric stabilization due to presence of large groups or existence of charged functional groups which leads to stabilization of the system as a result of electrostatic repulsion^32,33^. Thus, the synthesized CND is sterically stabilized due to existence of large groups like Rh6G hydrazide moiety covalently linked with citric acid. This can be easily confirmed by the insignificant change in time dependent absorbance value of the system under consideration (Fig. S13A,B). Further this stability was monitored using time dependent dynamic light scattering measurement where the counts of the scatterers were observed for a period of 48 h (Fig. S13C). No substantial changes were observed which corroborates the inherent stability of the prepared CNDs.

**Figure 3:**
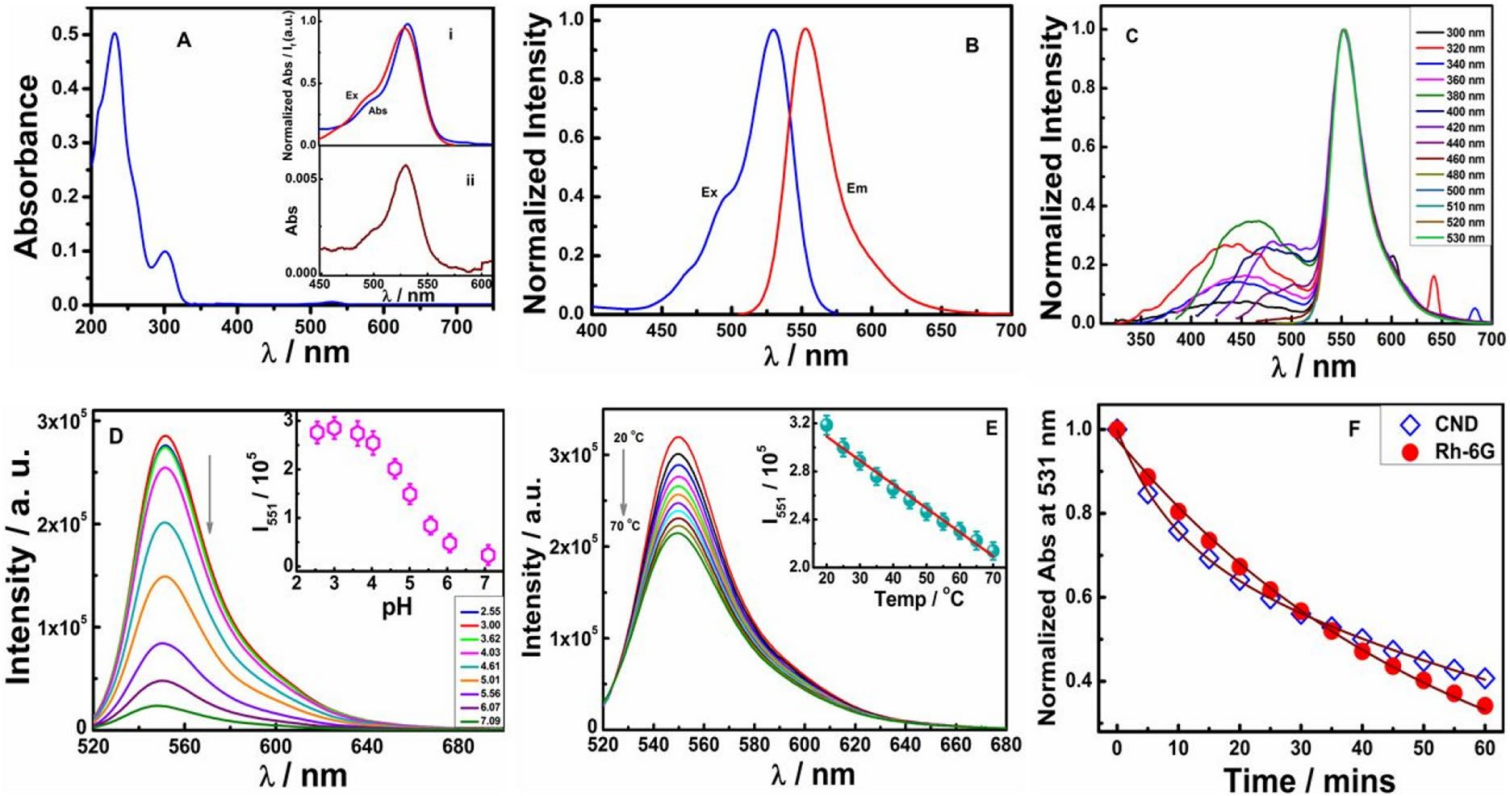
(**A**) UV/VIS absorption spectrum of CND (Inset (i) Normalized absorbance and excitation (ii) Magnified absorbance ~ 530 nm) (**B**) Excitation and emission spectrum of CND (**C**) Normalized PL of CND from 300 to 530 nm (**D**) pH dependent emission spectra of CND in aqueous solution (**E**) Temperature dependent emission spectra of CND in PBS buffer solution and (**F**) Normalized degradation plot of CND and Rh6G in ethanol. (I_551_ stands for fluorescence intensity at 551 nm and error limits for photo-physical experiments is ≤ 5%).

The UV–Vis absorption and excitation spectrum (Fig. 3A,B) exhibits a conspicuous absorption band at ~ 530 nm (indicating the presence of rhodamine moiety in CNDs) and at 230 nm, 300 nm and 400 nm which can be attributed to π–π* and n–π* transitions. Steady-state emission at different excitation wavelengths (from 300 to 530 nm) is shown in Fig. 3C.

Although excitation dependence for blue-emitting CNDs was reported in our earlier study^34,35^, the emission spectra of the current variant of CNDs were observed constantly at higher wavelength of 550 nm which is similar to observations made by Qu et al.^11^ where the CND exhibited strong orange luminescence with excitation independent PL in ethanol. The quantum yield of CND from citric acid and Rh6G hydrazide was found to be 0.90 (Figs. S14, S15) and this is the first report for orange CND for such a high quantum yield. For a better understanding of the origin of fluorescence of CND, pH dependent study of CND in 10% ethanolic aqueous solution was performed (Fig. S16). As evident from Fig. 3D, PL intensity of CND decreases progressively with increase in pH of the solution due to the reconstruction of spirolactam ring of Rh6G based moieties triggered by deprotonation of the amide group^15^. Thus, from the above study it can be inferred that the origin of PL properties of CND is solely the result of Rh6G hydrazide and citric acid based moieties which readily incorporate into CND structures and behave as molecular fluorophores. Initially, the aforementioned CND molecular fluorophores coalesce to form CND seeds forming core with characteristic emission at 300 nm and 400 nm. As a result of π–π stacking of molecular fluorophores it creates agglomerated larger CND structures as evident from AFM, TEM and SEM microscopy and orange fluorescence at 550 nm. Although the CND structure is bigger yet surface defects are not formed at all. This is shown by the high quantum yield of CND, otherwise such defects would primarily trigger fluorescence deactivation processes leading to decrease in PL. Therefore, such minimal or no defect can only arise if identical molecular fluorophores stack or self-assemble as observed in TEM images and WAXS study (Fig. S9B,C and Fig. S12B). Similar results were also derived by Langer et al.^36^ from their theoretical studies on citric acid based CNDs.

It has been observed that temperature affects both non-covalent interactions and conformational relaxation of molecules thereby significantly changing the photo-physical properties like emission intensity with change in temperature. For an increase in temperature, decrease in photoluminescence has been reported which can be ascribed to weakening of molecular interactions. Moreover, increase in temperature also speeds up thermal agitation in molecules which subsequently increases the molecular flexibility and enhances the non-radiative deactivation process^37,38^. Thus, temperature dependent study of CND (Fig. 3E) shows decrease in fluorescence intensity with the rise in temperature from 20 to 70 °C. This trend in decrease in PL is due to increase in molecular flexibility with the increase in temperature, leading to enhancement in non-radiative deactivation process. To ascertain the applicability of synthesized CND as a bio-imaging agent, photo-stability study of CND was carried out under intense Nd-YAG laser irradiation (λ_irr_ = 532 nm). It was found that CND exhibited better photo-stability as compared to Rh6G dye (Fig. 3F). Since temperature affects non-covalent interactions and conformational relaxation of molecular fluorophores, intracellular temperature dependence of CND fluorescence was hence studied^39^.

**Figure 4:**
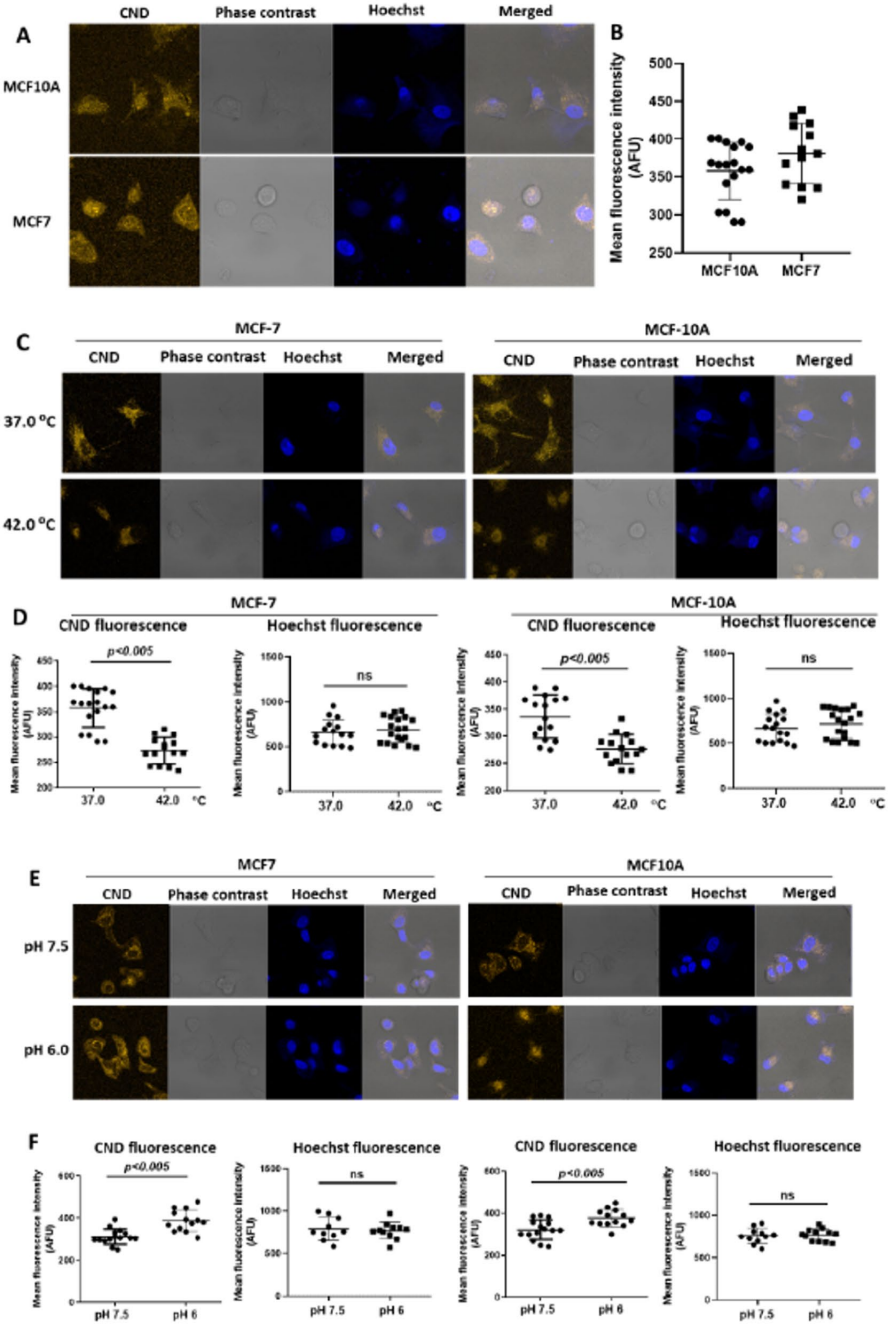
(**A**,**B**) Microscopic images (fluorescence and bright field) of MCF-7 and MCF-10 A cells, incubated with CND (5% vol./vol.) for 30 min. Quantification of fluorescence of cellular CND, in this experiment, is shown in (**B**). (**C**,**D**) Microscopic images (fluorescence and bright field) of MCF-7 and MCF-10 A cells, incubated with CND (5% vol./vol.) for 30 min and kept under different temperature (37 °C and 42 °C) for another 30 min. Quantification of fluorescence of cellular CND, in this experiment, is shown in (**D**). (**E**,**F**) Microscopic images (fluorescence and bright field) of MCF-7 and MCF10-A cells, incubated with CND (5% vol./vol.) for 30 min and kept under different pH (6.0–7.5) Quantification of fluorescence of cellular CND, in this experiment, is shown in (**F**). (AFU stands for arbitrary fluorescence unit).

Extensive studies have demonstrated hyperthermia (heating < 43 °C for 30–60 min) improve the outcomes of chemotherapy and radiotherapy of cancer^40^. However it is imperative to probe the intracellular temperature to understand the biological effects of hyperthermia at high temperature. Intracellular pH (pH_i_ = 7.0–7.2) in normal cells is lower than extracellular pH (pH_e_ = 7.3–7.4). In contrast, cancer cells have higher values of pH_i_ (7.12–7.25) and lower pH_e_ (6.2–6.9). Reversal in the pH gradient assists in the progression of cancer such that elevated pH_i_ allows cancer cell proliferation^41^. During short duration of hyperthermia (30–60 min), it is expected to have no effect on the intracellular pH_i_, which requires long term switching effects of glycolysis. In this regard, incubation of CND with MCF-7 (breast adenocarcinoma) led to rapid uptake and cytoplasmic accumulation of CND, as evident from the high orange fluorescence of CND (Fig. 4). In order to ascertain selectivity towards cancer cells against normal cells of same origin, intracellular temperature or pH variation were studied in MCF-10A (human mammary epithelial cell line) which are normal cells of breast origin (Fig. 4). The experiments with CND were also carried out along with Hoechst fluorescent dye as a control counter stain. The results showed that, although variation in fluorescence of CND was correlated with change in temperature and pH, no such change was observed for Hoechst dye (Fig. 4). Hence, our results suggested the specificity of CND towards variation in intracellular temperature and pH. However, no selectivity was observed towards MCF-7 as compared to MCF-10A. Interestingly, progressive increase in the temperature from 37 to 42 °C (Fig. S16), significantly reduced PL of CND in a temperature dependent manner. Strikingly, our result showed that a small change in the cellular temperature (37–39.5 °C) is well detected by CND (Fig. 4C,D). Moreover, intracellular pH dependent study of CND revealed that PL intensity of CND decreases with the increase in pH from 6.0 to 7.5 in MCF-7 (Fig. 4E,F). This indicated CND fluorescence sensitivity to intracellular pH_i_. In order to evaluate whether Rh6G-CND remains intact or dissociated in intracellular environment, characteristic fluorescence emissions of Rh6G-CND (Fig. S19) was assessed in MCF-7 cells by using lambda mode scanning in confocal microscope. Upon excitation with 355 nm laser, intracellular fluorescence of the dye was analyzed at 412–657 nm (at the interval of 5 nm). Our result revealed that fluorescence emission spectra of CND, at three random locations inside cells, were quite similar to that of Rh6G-CND (Figs. S19 and S20) suggesting that Rh6G-CND dye remains intact under intracellular environment to act as a nano-sensor. Besides, long term incubation of CND (5% vol./vol. for 72 h) with MCF-7 cells, did not cause loss of cell viability, as assessed by the MTT assay (Fig. S21). To evaluate biocompatibility and non-toxicity of CNDs, MTT assay^42^ was performed in both normal and cancer cells namely CHO (Chinese hamster ovary cell line) and MCF-7 cells respectively. IC_50_ value in CHO cells was 1133 µg/mL while in MCF-7 cells; IC_50_ of 1177 µg/mL was observed (Fig. S22).

## Conclusions

This is the first report of highly fluorescent (QY of 90%) Rh6G and citric acid based CND, achieved from a facile and solvent-free synthesis route. The synthesized CND was easily purified using dialysis and the dialysate was further studied for its photo-physical and particle size properties and bio-imaging application. The CND behaves as a molecular fluorophore which is susceptible to temperature variations and the spirolactam ring present in the CND is pre-disposed to changes in pH. The dual sensor attributes for temperature and pH sensing makes CND ideal as a bio-imaging agent for cancer related studies. The results clearly show that CND is non-toxic to both normal and cancer cells. Together, synthesized CND can be used as a sensitive bio-imaging probe to measure intracellular temperature and pH with no or minimum toxicity and excellent biocompatibility.

## Methods

Infrared spectroscopy was carried out in Bruker TENSOR III instrument. The measurement was made by attenuated total reflection method with frequency range of 4000–500 cm^−1^. ^1^H and ^13^C NMR was recorded in VARIAN 500 MHz and Bruker 300 MHz instrument. The SCXRD data were collected from a single crystal at 298(2) K on a XtaLAB Synergy, Dualflex, HyPix four-circle diffractometer with a micro-focus sealed X-ray tube using mirror as monochromator and a HyPix detector. Elemental analysis was carried out using Eurovector Instruments and Software EuroEA3000 CHNS-O elemental analyzer instrument. Scanning electron microscopy was performed using SEM-Carl Zeiss, Germany coupled with Dispersive X-ray spectroscopy (EDX)-INCA Energy 250, Oxford Instruments. Sample was prepared on a clean glass slide and sputtered with gold using standard methods. The scanning of images was performed at an accelerating voltage of 10 keV and EDX spectra were obtained between 0 and 20 keV.

### Synthesis of Rh6G hydrazide

Rh6G (5 g, 10.4 mmol) was dissolved in methanol (50 mL) and to the stirred solution hydrazine hydrate (1 g, 30 mL) was added. The reaction mixture was refluxed for 6 h and then allowed to cool to room temperature. To the resulting solution, ethyl acetate was added and washed with 33 mL of NaOH (1 M) solution twice. The obtained Rh6G hydrazide was dried over MgSO4 and purified using silica gel chromatography (25% MeOH: 75% CHCl3). Recrystallization was done in chloroform and methanol solvent system and colorless crystals were obtained (4 g, 90% yield) which was characterized by FT-IR, ^1^H NMR (Fig. S1), ^13^C NMR (Fig. S2) and single crystal XRD (CCDC 2051200, Fig. S3)^22,23^.

- *FT-IR*: 3427, 2973, 2872, 1680, 1621, 1514, 1463, 1420, 1344, 1268, 1201, 1154, 1009, 834, 773 cm^−1^.
- ^*1*^*H NMR (500 MHz, DMSO-d*_*6*_*)*: *δ* 7.78–7.73 (m, 1 H), 7.49–7.44 (m, 2 H), 6.95–6.91 (m, 1 H), 6.27 (s, 2 H), 6.10 (s, 2 H), 3.13 (q, 4 H, *J* = 3.5 Hz, *J* = 11 Hz), 1.87 (s, 6 H), 1.20 (t, 6 H, *J* = 17.5 Hz).
- ^*13*^*C NMR (300 MHz, DMSO-d*_*6*_*)*: 14.2, 17.1, 37.5, 65.1, 96.0, 105.1, 117.9, 122.2, 123.5, 127.1, 128.1, 129.5, 132.4, 147.4, 151.4, 152.2, 165.4.

### Synthesis of CND

Citric acid (3 g) was heated at 200 °C to give a colorless melt in which Rh6G hydrazide (0.01 g) was added resulting in immediate color change from colorless to purple. The resulting liquid was heated at 200 °C for ten minutes, allowed to cool down to room temperature and dissolved in 50 mL of ethanol which gave an orange-colored fluorescent solution. The solution was put in a dialysis bag and dialyzed for 6 h. The resulting dialyzed solution was characterized by ^1^H NMR (Fig. S4), ^13^C NMR (Fig. S5), FT-IR (Fig. S6), elemental analysis (Table. S5), EDX (Fig. S7), AFM (Fig. S8), TEM (Fig. 2D, Fig. S9), UV–VIS and Fluorescence spectroscopy (Fig. 3).

- *FT-IR*: 3494.5, 2988.8, 2535.4, 1842.2, 1701.6, 1633.6, 1400.5, 1186.7, 973.5, 893.7, 825.8, 784.9, 729.4, 697.8, 589.5 cm^−1^.
- ^*1*^*H NMR (300 MHz, MeOH-d*_*4*_*)*: *δ* 6.90 (s), 6.81 (d, *J* = 3 Hz), 6.28 (s), 5.89 (d, *J* = 3 Hz), 5.74 (s), 3.31(s), 2.94–2.76 (m), 2.14 (s), 2.05 (d, *J* = 3 Hz), 1.36–1.22 (m).
- ^*13*^*C NMR (300 MHz, MeOH-d*_*4*_*)*: 9.8, 11.4, 20.5, 20.6, 20.9, 27.14, 33.83, 36.6, 38.4, 42.1, 43.9, 74.3, 121.9, 122.5, 129.2, 129.6, 130.6, 130.7, 136.3, 147.1, 147.7, 150.8, 166.1, 168.1, 168.2, 168.5, 169.8, 172.6, 172.8, 173.6, 173.6, 175.0, 176.9.

### Synthesis of citric acid CND

Citric acid (3 g) was heated at 350 °C for fifteen minutes to give a colorless melt which turned into sticky yellow compound. The product was allowed to cool down to room temperature and dissolved in 50 mL ethanol which gave a pale yellow solution. The solution was put in dialysis bag and dialyzed for 6 h. The resulting dialyzed solution was characterized by UV–VIS and Fluorescence spectroscopy (Figs. S18 and S19), ^1^H NMR (Fig. S23), ^13^C NMR (Fig. S24), FT-IR (Fig. S25) and

- *FT-IR*: 3108.8, 2942.1, 1845.9, 1767.3, 1703.4, 1404.2, 1177.2, 992.7, 892.5, 775.8, 721.6, 628.2 cm^−1^.
- ^*1*^*H NMR (300 MHz, MeOH-d*_*4*_*)*: *δ* 7.06 (s), 6.90 (s), 6.73 (s), 6.39 (s), 6.28–6.24 (m), 5.88 (s), 5.74 (s), 3.88 (s), 3.65–3.61 (m), 3.07–2.76 (m), 2.16 (s), 1.30 (m).
- ^*13*^*C NMR (300 MHz, MeOH-d*_*4*_*)*: 24.3, 31.5, 34.7, 38.4, 39.8, 41.9, 44.0, 74.3, 128.4, 129.0, 129.4, 132.7, 136.5, 139.2, 165.7, 169.3, 171.1, 173.6, 176.9.

The data were collected from a single crystal at 298(2) K on a XtaLAB Synergy, Dualflex, HyPix four-circle diffractometer with a micro-focus sealed X-ray tube using mirror as monochromator and a HyPix detector. The diffractometer was equipped with a low temperature device and used Cu *K*_*α*_ radiation (λ = 1.54184 Å). All data were integrated and a multi-scan absorption correction was applied using CrysAlis PRO^24^. The structure was solved by direct method using OLEX and refined by full-matrix least-squares method against *F*^2^ by SHELXL-2017/1^25,26^. Hydrogen atoms were placed in idealized positions and were set riding on the respective parent atoms. All non-hydrogen atoms were refined with anisotropic thermal parameters. Crystallographic data (including structure factors) for the structure reported in this paper have been deposited with the Cambridge Crystallographic Data Centre (CCDC No. 2051200). Oakridge thermal ellipsoid plot (ORTEP) was employed for the final data presentation and structure plots^27^. NTMDT Ntegra Atomic Force Microscope was used for AFM. About 10 µL of the CND solution was spotted on to freshly cleaved mica surface and allowed to air dry in laminar air flow for about 25–30 min. This mica adhered CND sample was attached to sapphire substrate and placed on the sample holder of NTMDT Ntegra Atomic Force Microscope. The mica surface was scanned in semi contact mode with NSG11 golden silicon probe tip using a 10-micron scanner under universal head. Measurements across X–Y–Z axis were measured using the NTMDT NOVA 1.1.01780 software. Arbitrary height measurement tool of this software was used to measure height of 50 randomly selected CNDs as described earlier^28^.

Transmission Electron Microscopy was carried out in LIBRA 200FE TEM instrument. TEM samples were prepared by evaporating 10 µL of sample solution on carbon coated (Carbon Type-B, 200 mesh) Cu grids purchased from TED PELLA Inc. Small angle X-ray scattering (SAXS) was done in Anton Paar SAXSpace instrument which uses line collimated monochromatic X-ray source (Cu *K*_*α*_, λ = 0.1542 nm) operated at 40 kV and 50 mA. The sample was placed in paste cell and thermostated using Peltier controlled sample holder. The scattering intensities were monitored by using a two dimensional (2D) charged-coupled device (CCD) detector (pixel size 24 micron) to span q (momentum transfer) range of 0.15 to 5 nm^−1^. A semi-transparent beam stop was employed to measure transmittance and zero q position. The 2D SAXS images were processed into one-dimensional (1D) scattering profiles and were collected for transmission and background scattering using standard protocols in SAXSquant (Anton Paar, Austria) software. Ensemble averaged microstructural analysis of the carbon dots was carried out by SAXS. Fig. S12A shows the background subtracted SAXS pattern of the CND which reveals the amorphous nature with spatial inhomogeneity in the electron density, as reflected from an increase in the scattering at lower momentum transfer (q) values. For a polydispersed population of spherical particles, the scattering pattern can be computed as a sum of the scattering from individual size particles, I_p_ (q, R) weighted by the distribution function, as given by the equation

$$I\left( {q, R} \right) \propto \mathop \smallint \limits_{0}^{{R_{max} }} D_{v} \left( R \right)R^{3} I_{P} \left( {q, R} \right)dR$$

$$I_{p } \left( {q, R} \right) = K \cdot \rho^{2} V_{P} \left( {\frac{{\sin \left( {qR} \right) - qRcos\left( {qR} \right)}}{{\left( {qR} \right)^{3} }}} \right)^{2}$$

where D_v_(R) represents the volume weighted particle size distribution, K is a constant depending on the instrument geometry, Δρ is the scattering contrast given by difference in the electron density of matrix and particle, and V_p_ is the particle volume. Figure 2F shows the corresponding distribution of carbon dots obtained by fitting the scattering curve using indirect Fourier transform (IFT) software. The distribution shows that CND having particles in the range mostly from 1.5 to 15 nm^43^.

Wide angle XRD X-ray scattering (WAXS) was done in Rigaku Smart Lab (9 W) Wide angle XRD instrument. The experiment was performed with a scan from 5 to 80° with 3° per minute at 6 W power. The zeta-potential measurement was carried out in Zetasizer nanoseries, Malvern Instruments. A UV–Vis spectrophotometer (Model UV-2700, Shimadzu, Japan) was used to carry all the ground-state absorption measurements in an optical quartz cell of path length 1 cm. The steady-state (SS) fluorescence measurements for the studied systems were carried out using spectrofluorometer (Model Fluoromax-4, Horiba, UK) where sample solutions were taken in a 10 mm × 10 mm quartz cuvette and excited with 515 nm steady light beam. In the temperature dependent study, the sample solutions were heated using a temperature controller (Model 35B, Newport, USA) having accuracy of ± 0.5 °C. The temperature was increased in a stepwise manner, changing in the steps of 5 °C, and allowing the solution to equilibriate at each set temperature for about 10 min, before taking the measurements. The adjustment of the pH was done using dilute solutions of NaOH and HCl and measured using edge blu Bluetooth smart pH meter-HI 2202. All the photo-physical studies were carried out at a concentration of ~ 0.3 mg/mL of CND. Photo-degradation studies were carried out using Nd: YAG nanosecond laser (M/s Ekspla) with pulse duration 6 ns, repetition rate 10 Hz at 532 nm wavelength emission. Dialysis purification was done in MilliQ water using Spectra/Por Molecular Porous Membrane Tubing membrane MWCO:12-14000. Rhodamine 6G, citric acid and hydrazine hydrate were purchased from Sigma-Aldrich. Quantum Yield experiment was carried out using relative quantum yield method. Rhodamine 6G was taken as a standard (QY = 0.95 at 505 nm). The measurements of the CND sample were carried out in ethanol and QY obtained was 90% at 505 nm.

MCF-7 (human breast cancer) and MCF-10 A cell lines were purchased from ECACC (Europe) while CHO cells were purchased from National Centre for Cell Sciences, Pune, India and maintained in high glucose containing Dulbecco’s Modified Eagle Medium (DMEM) (Himedia, India) at 37 °C under 5% CO_2_. MCF-7 cells (50,000 cells/well) were seeded in 35-mm imaging dish (genetix) and incubated overnight. The cells were gently washed with DMEM media and maintained at respective temperatures for 10 min, followed by imaging. Cellular imaging was performed in live-cell mode of LSM 780 confocal microscope (Zeiss) with inbuilt temperature control module (excitation 488 nm, emission 580–630 nm). T-PMT was used to capture bright field images of the cells. The lambda scanning for fluorescence emission spectra of the intracellular CND was carried out with confocal microscope (Zeiss LSM 780). All chemicals and reagents like MTT (3-(4,5-Dimethyltiazol-2-yl)-2,5-diphenyltetrazolium bromide), sodium acetate, sodium chloride, sodium bicarbonate, Dimethylsulfoxide (DMSO), 4-(2-hydroxyethyl)-1-piperazineethanesulfonic acid (HEPES), DMEM media were procured from Sigma chemical Inc. (USA). Fetal Bovine Serum (FBS) was purchased from GIBCO Laboratories, USA. Polar star Omega Plate Reader Spectrophotometer-BMG LABTECH, Germany was used to measure spectrometric reading. The IC_50_ values were calculated using graphpad prism 6. MTT (3-(4, 5-dimethylthiazol-2-yl)-2, 5-diphenyltetrazolium bromide) assay, a colorimetric assay was employed to measure cellular metabolite activity and hence the cell viability. Viable cells that contain NAD(P)H-dependent oxidoreductase enzymes reduce the MTT reagent to formazan, to give a deep purple color. For MTT cytotoxicity assay, MCF-7 (Human breast adenocarcinoma cell line) and CHO (Chinese Hamster ovary cell line) cells were cultured in DMEM medium containing 10% FBS and grown up to 60–70% confluence at 37 °C and 5% CO_2_ in an incubator. After trypsinization, 7 × 10^3^ cells were seeded in 96 well tissue culture plates and allowed to grow monolayer after incubation overnight at 37 °C and 5% CO_2_. MCF-7 and CHO cells were treated with different concentrations of carbon nanodots (18.75–25,298 µg/mL) and incubated for 18 h. Vehicle control was set up wherein cells were treated with formulation without carbon nanodots. Control cells were incubated in cell culture medium. All treatments were carried out in quadruplicate (n = 4). After 24 h, MTT solution (5 mg/mL in Phosphate Buffered saline (PBS)) was added to each well for dissolving the formazan crystals. The plate was kept on plate shaker in dark. After 30 min, absorbance was measured at 570 nm that corresponds to viability of cells. Absorbance at 600 nm was used as a reference wavelength. The percentage of dead cells was calculated as ([optical density (OD) of vehicle control sample − OD of treated sample/OD of control sample] * 100) and represented as mean ± Standard Deviation (SD) (n = 4). This was plotted against different log concentrations of the CND formulation and half maximal inhibitory concentration (IC_50_) was calculated. MCF-7 cells were incubated with 1075.6 µg/mL of CND for 24 h for its internalization. After 24 h, cells were washed with PBS, pH 7.4 and acid stripping buffer (DMEM with 0.2% bovine serum albumin (BSA), pH 3.5) alternatively twice before suspending cells in 1 mL PBS, pH 7.4. This procedure was carried out to remove supernatant and membrane bound CND in the cells after internalization. The photoluminescence intensity (PL) was measured at excitation 544 nm and emission 590 nm, with 10 °C increase in temperature from 25 to 45 °C.

## Supplementary Information

Supplementary Information.
