# Supplementary material for: Carbon nano-dot for cancer studies as dual nano-sensor for imaging intracellular temperature or pH variation
Source: Sci Rep. 2021 Dec 21;11:24341. doi: 10.1038/s41598-021-03686-x (PMC8692618; doi:10.1038/s41598-021-03686-x)
Supplement: Supplementary file 1 — Supplementary Information. [file 41598_2021_3686_MOESM1_ESM.pdf]

Supplementary Information

Carbon Nano-dot for Cancer Studies as Dual Nano-Sensor for Imaging Intracellular Temperature or pH variation

Trilochan Gadly,[1*] Goutam Chakraborty,[2] Mrityunjay Tyagi,[1] Birija S. Patro, [1] Bijaideep Dutta, [3] Akhilesh Potnis, [4] Pallavi Chandwadkar,[4] Celin Acharya,[4] Shishu Kant Suman,[5] Archana Mukherjee,[5] Suman Neogy,[6] Amey Wadawale,[3] Srikant Sahoo,[7] Nitish Chauhan,[1] Sunil K. Ghosh[1]

[1] Bio-Organic Division, [2]Laser & Plasma Technology Division, [3]Chemistry Division, [4]Molecular Biology Division, [5]Radiopharmaceuticals Division, [6]Material Science Division, [7]Analytical Chemistry Division, Bhabha Atomic Research Centre, Trombay, Mumbai-400085, India

Correspondence to: tgadly@barc.gov.in

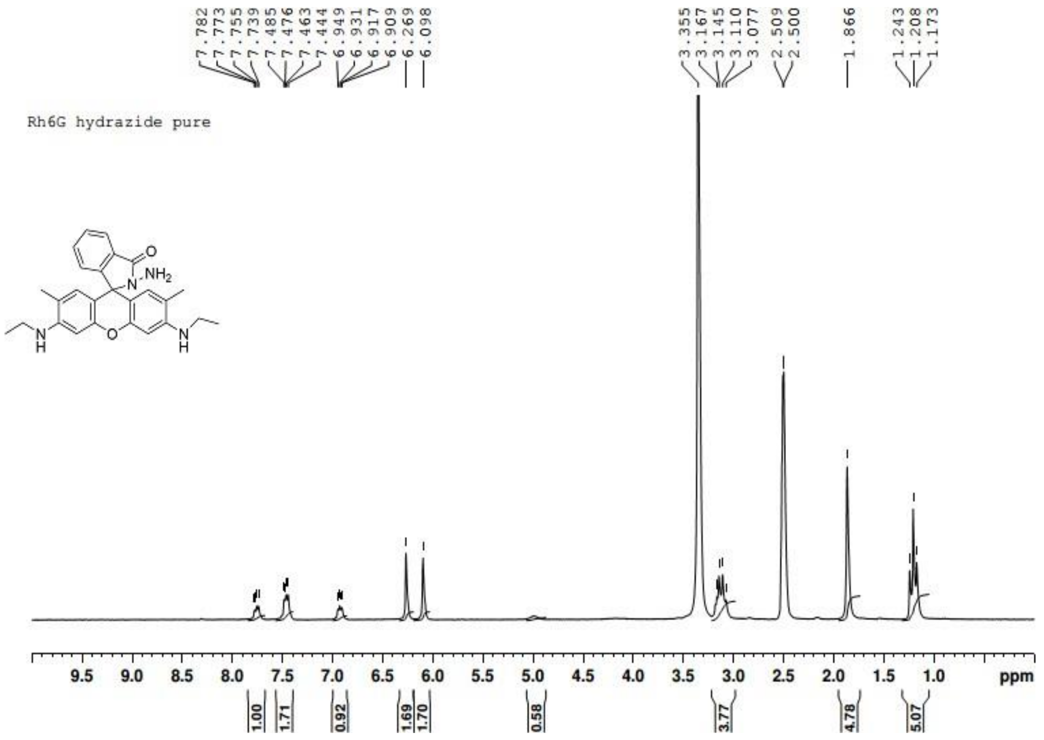

Fig. S1: ^{1}H NMR of Rh6G hydrazide.

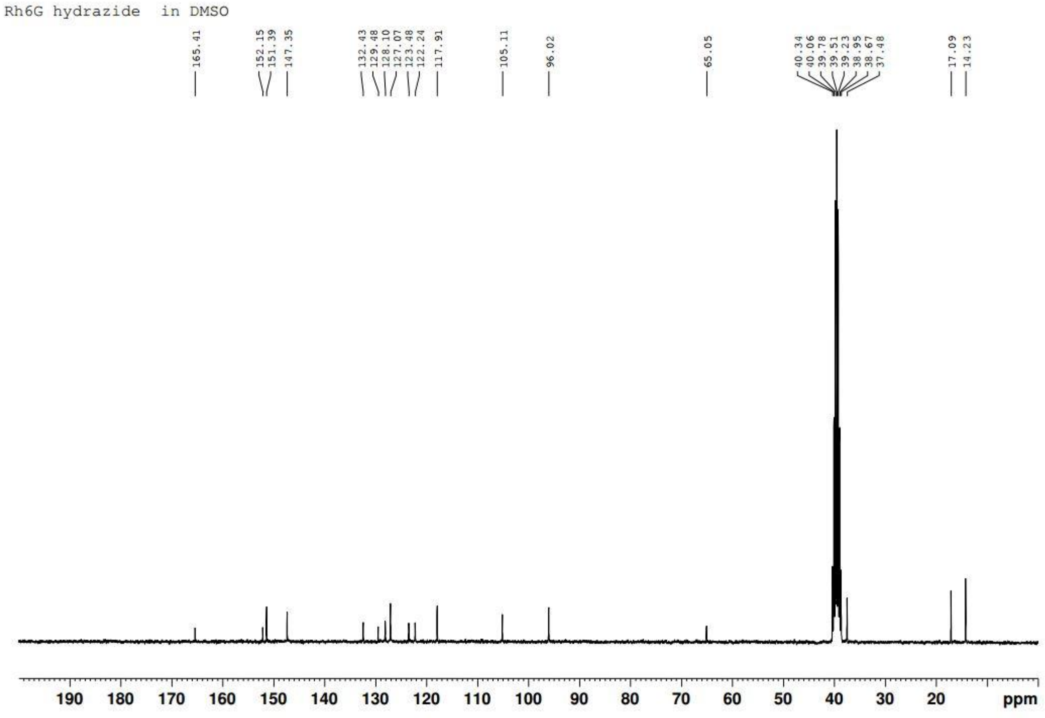

Fig. S2: ^{13}C NMR of Rh6G hydrazide.

Single Crystal XRD of Rh6G hydrazide

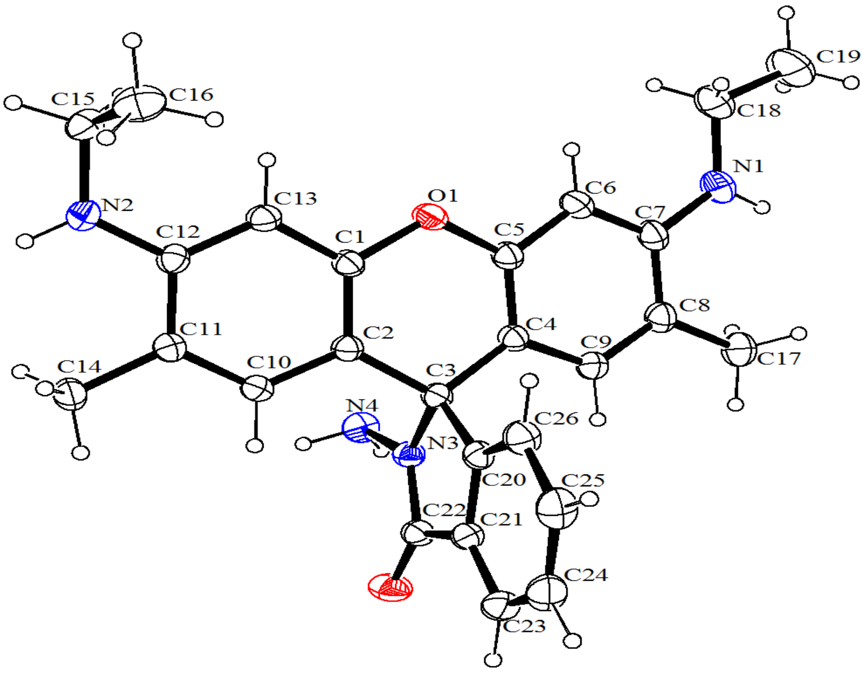

Fig. S3. Thermal Ellipsoid plot of Rh6G hydrazide at 25% probability.

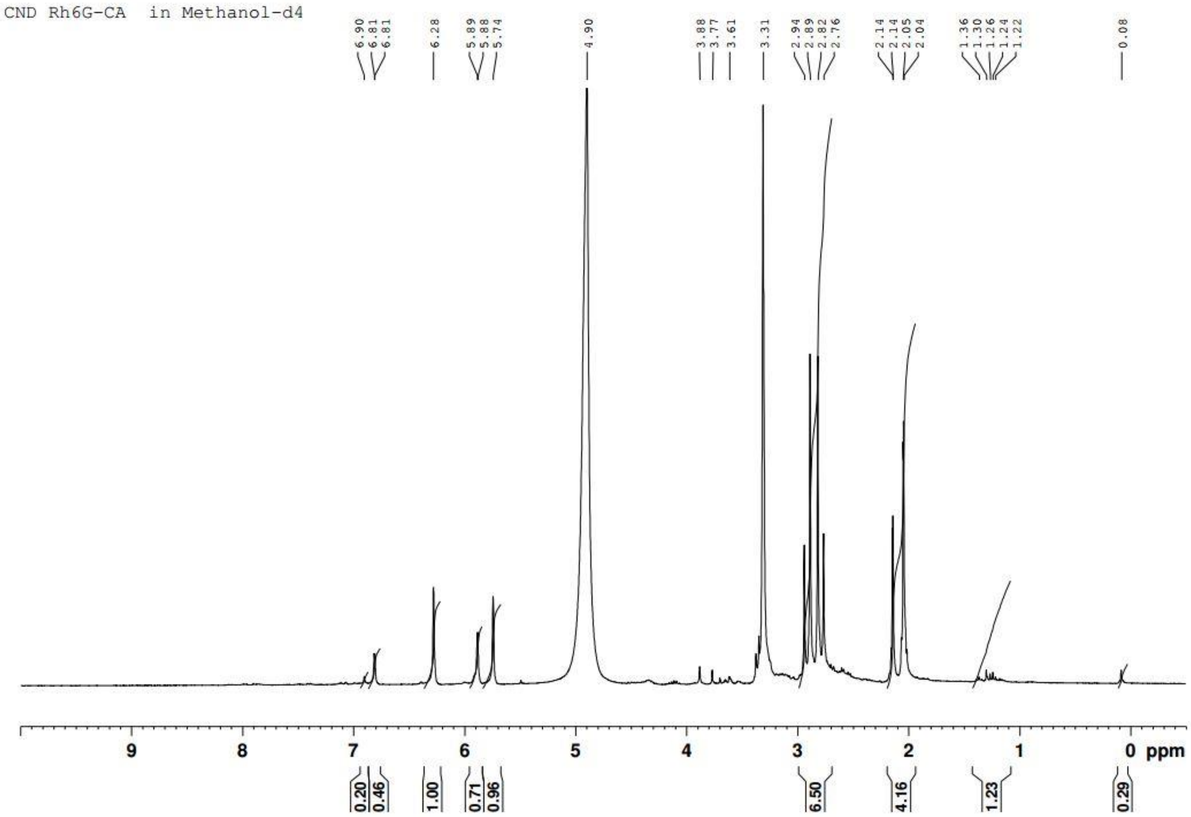

Fig. S4: ^{1}H NMR of CND.

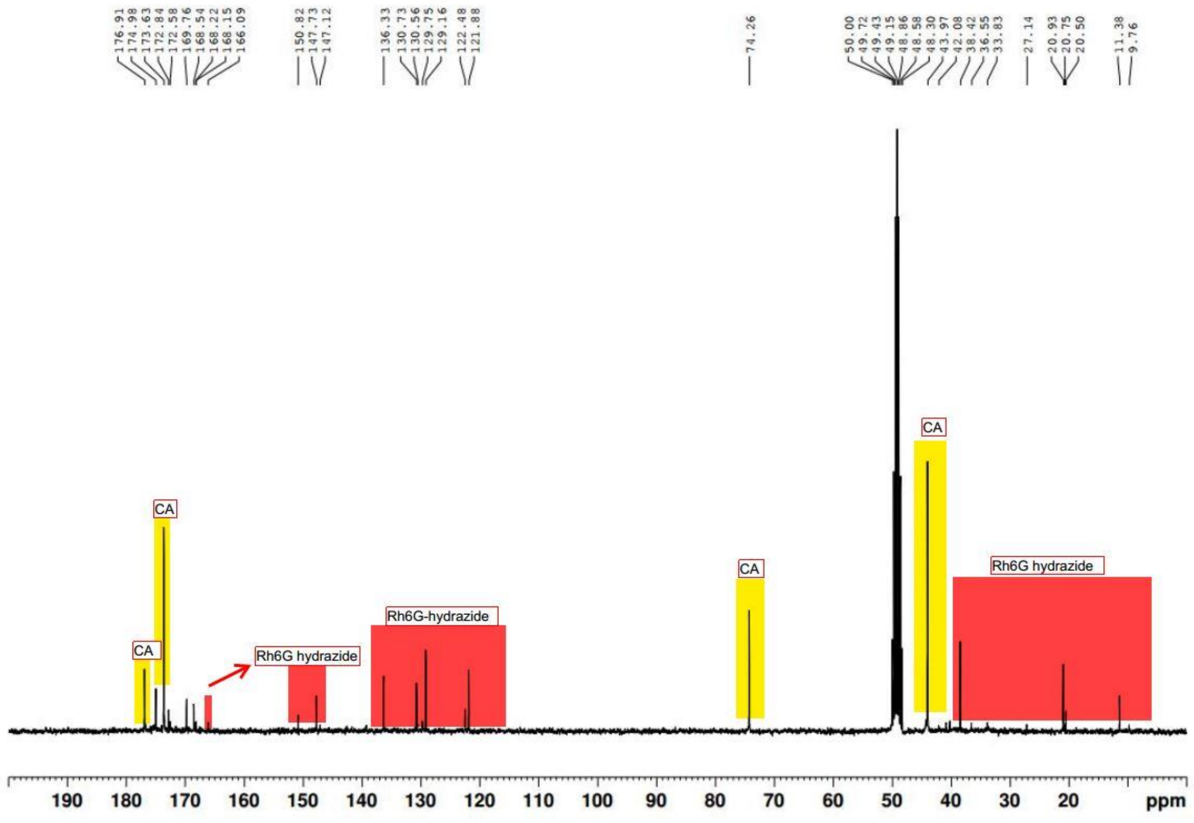

Fig. S5: ^{13}C NMR of CND (where CA = Citric acid and Rh6G = Rhodamine 6G).

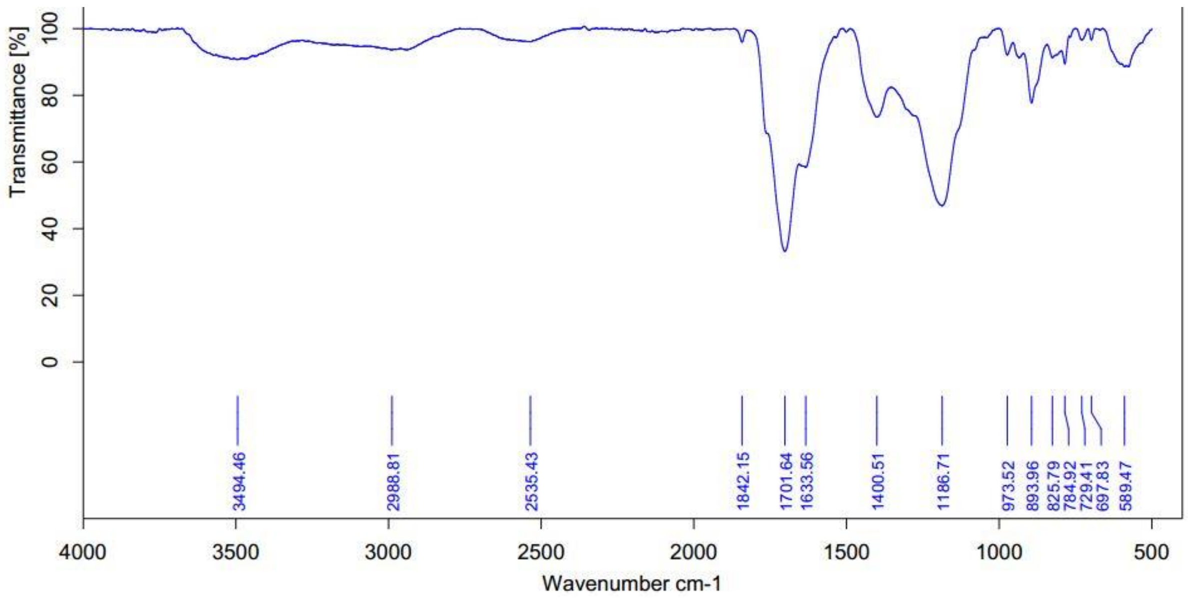

Fig. S6. FT-IR spectrum of CND.

Viewed Data: Spectrum 8

Result Type: Weight %

Processing Option Used: All Elements Processed (Normalized)

Element	Wt%	Wt% Sigma
C	51.28	0.78
O	41.04	0.74
Na	0.90	0.08
Si	5.00	0.13
Ca	1.28	0.07
K	0.27	0.05
Mg	0.24	0.05
Total	100.00	

Fig. S7. Elemental composition of CND by EDX spectroscopy

Atomic Force Microscopy (AFM)

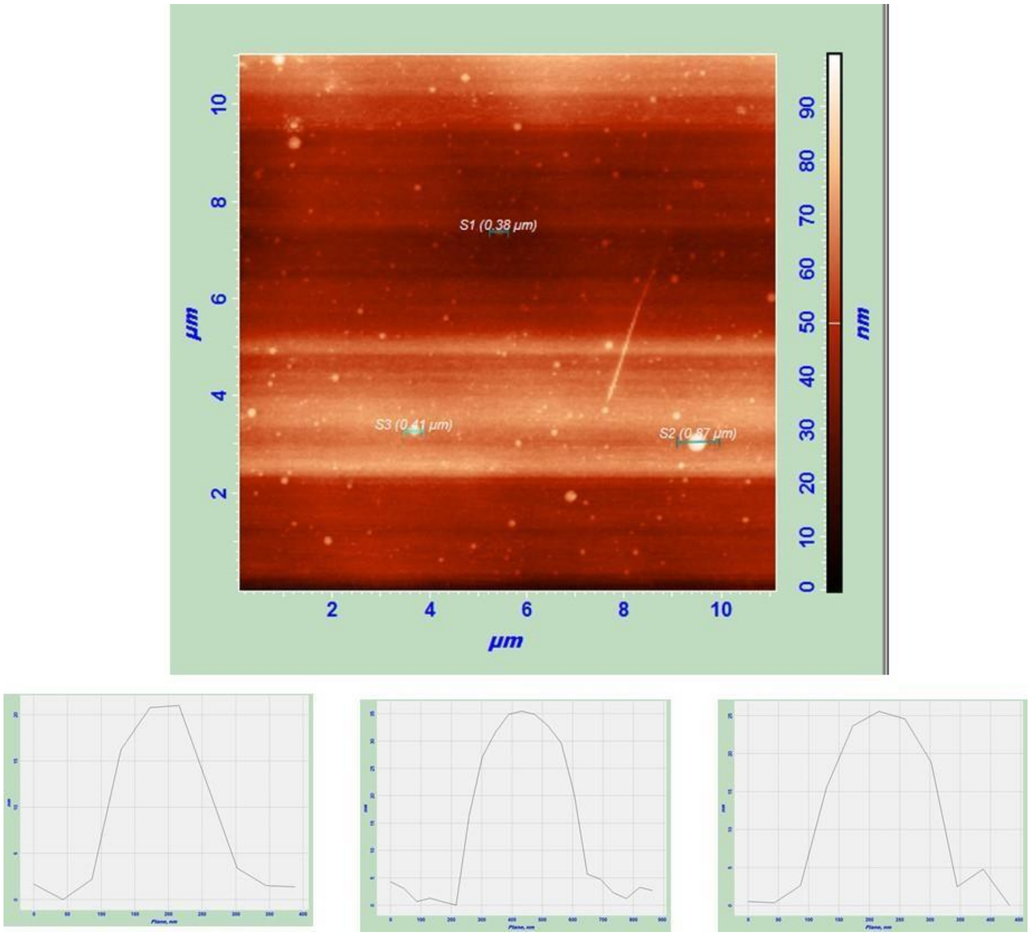

Fig. S8. A) AFM of CND **B)** Height profiles of CND particles

Transmission Electron Microscopy (TEM)

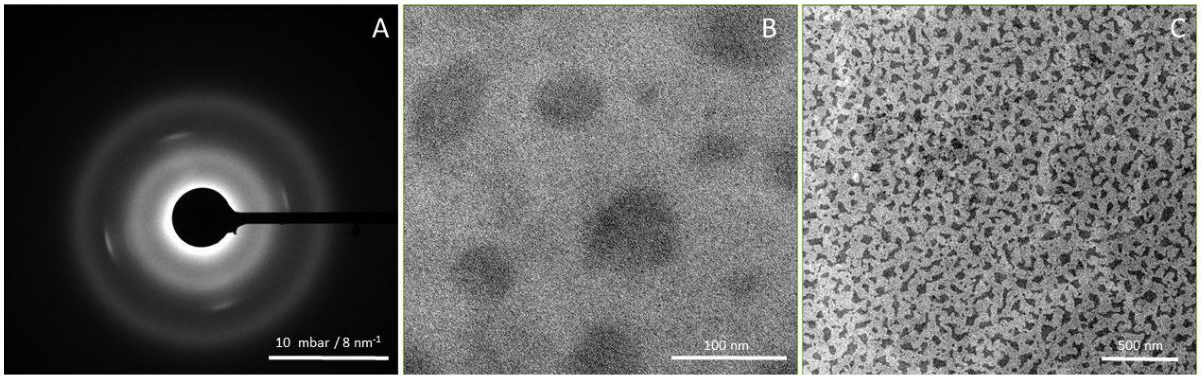

Fig. S9. TEM of CND **A)** Selected area electron diffraction pattern showing amorphous nature of CND **B**) Higher magnification image showing lack of core-shell structure **C)** Agglomeration in CNDs.

Scanning Electron Microscopy (SEM)

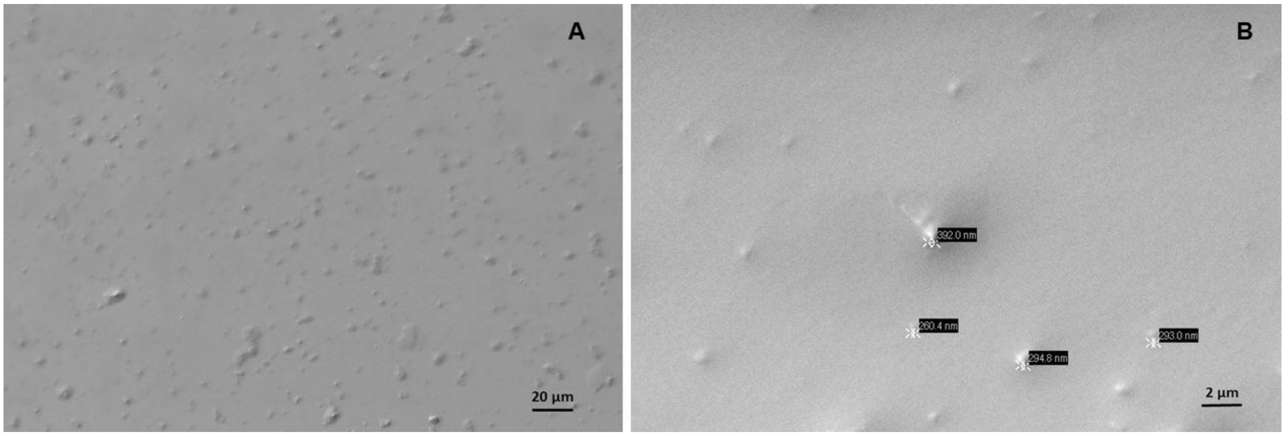

Fig. S10. SEM of CND.

Zeta Potential

Record Number:	6	Dispersant RI:	1.330
Date and Time:	Thursday, December 10, 2020 3:22:29 AM	Viscosity (cP):	0.8872
		Dispersant Dielectric Constant:	78.5

Temperature (°C):	25.1	Zeta Runs:	5
Count Rate (kcps):	73.6	Measurement Position (mm):	0.00
Cell Description:	Zeta dip cell	Attenuator:	9

			Mean (mV)	Area (%)	Width (mV)
Zeta Potential (mV):	1.12	Peak 1:	0.947	99.7	7.57
Zeta Deviation (mV):	8.20	Peak 2:	58.5	0.3	6.74e-7
Conductivity (mS/cm):	0.979	Peak 3:	0.00	0.0	0.00

Result quality : See result quality report

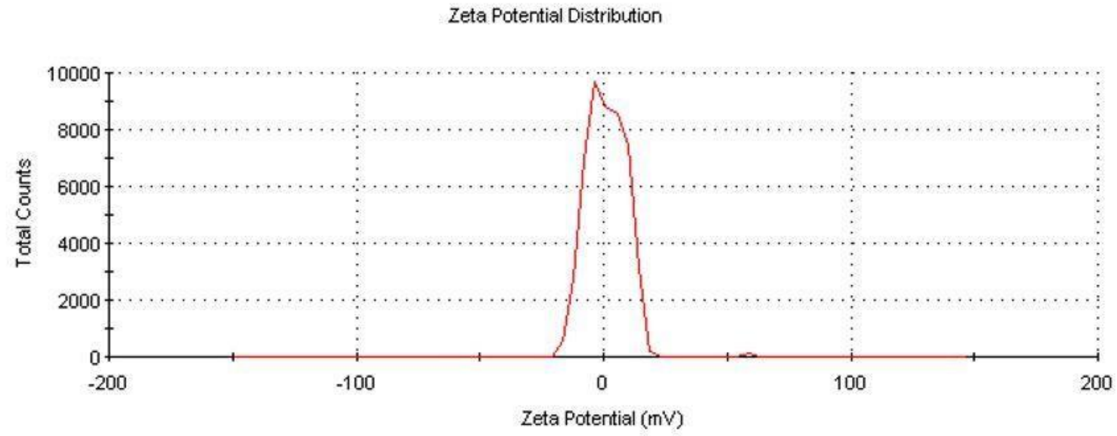

Fig. S11. Zeta Potential Distribution Graph

Small Angle X-ray Scattering (SAXS) and Wide Angle Scattering (WAXS) Experiments

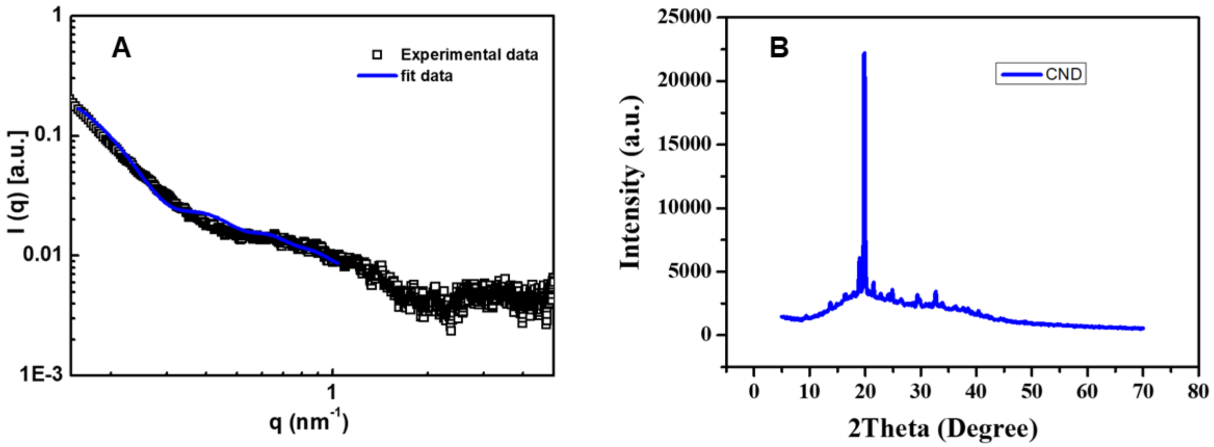

Fig. S12. A) SAXS intensity pattern of amorphous CND (solid blue line shows the model fit) **B**) Wide Angle X-ray scattering (WAXS) spectrum of CND showing both crystalline and amorphous nature.

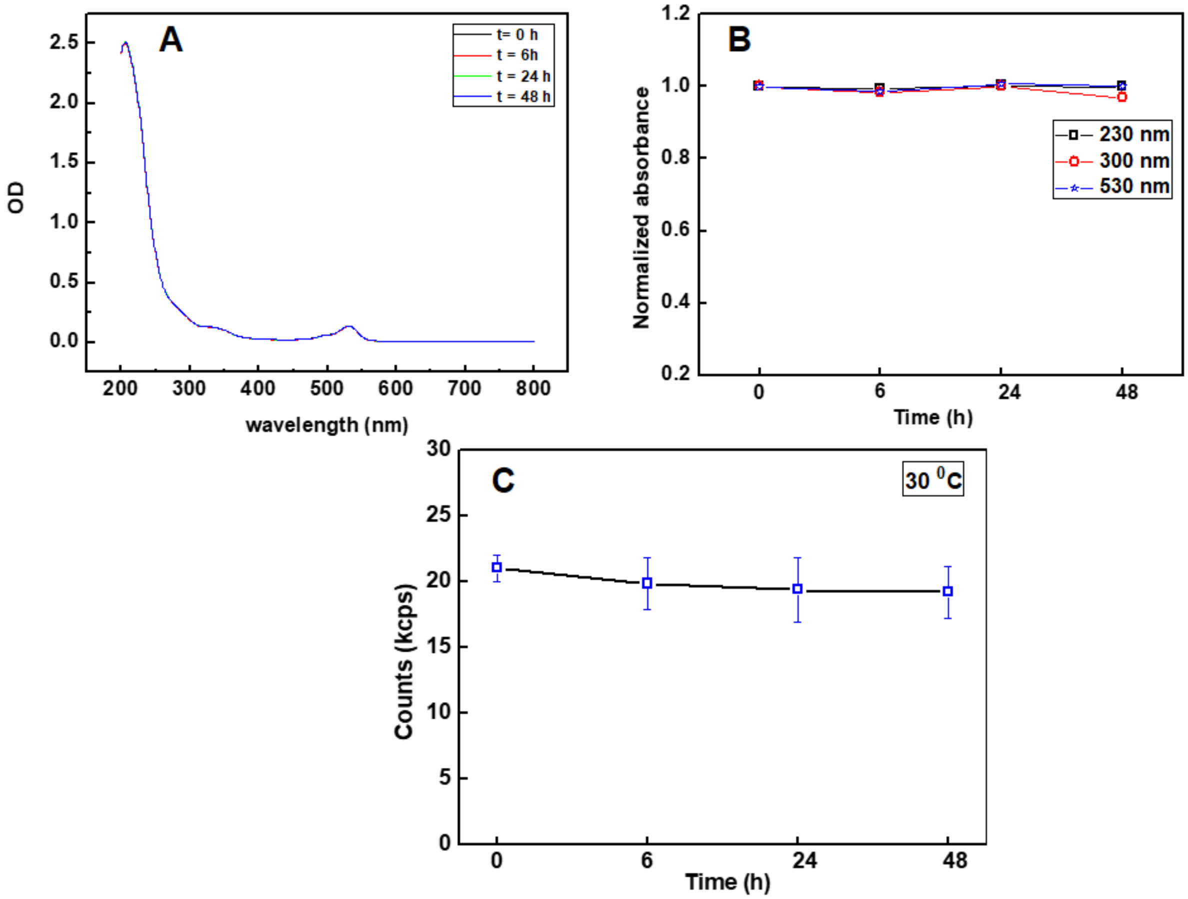

Fig. S13. A) Time dependent absorbance of the CND in water **B)** Normalized absorbance graph for the system in different time scale **C)** Time dependent DLS count rate.

Quantum Yield Determination

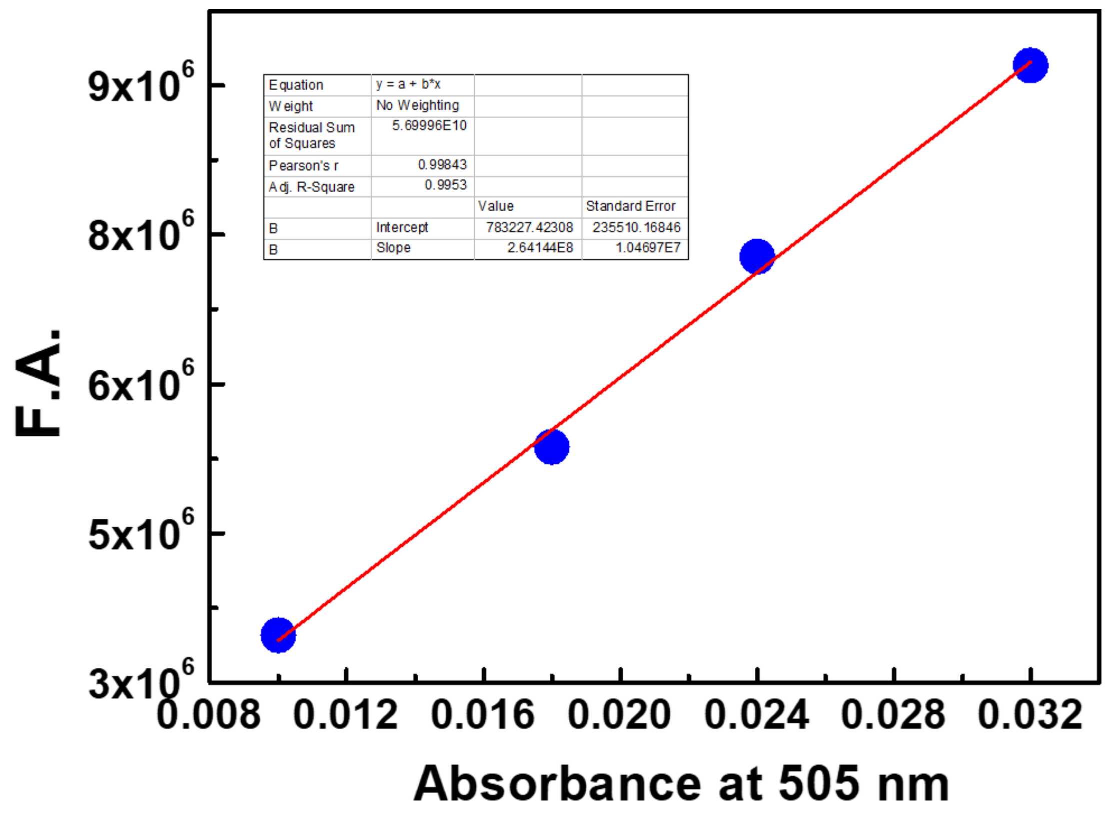

Fig. S14. Gradient plot for CND

Quantum Yield Determination

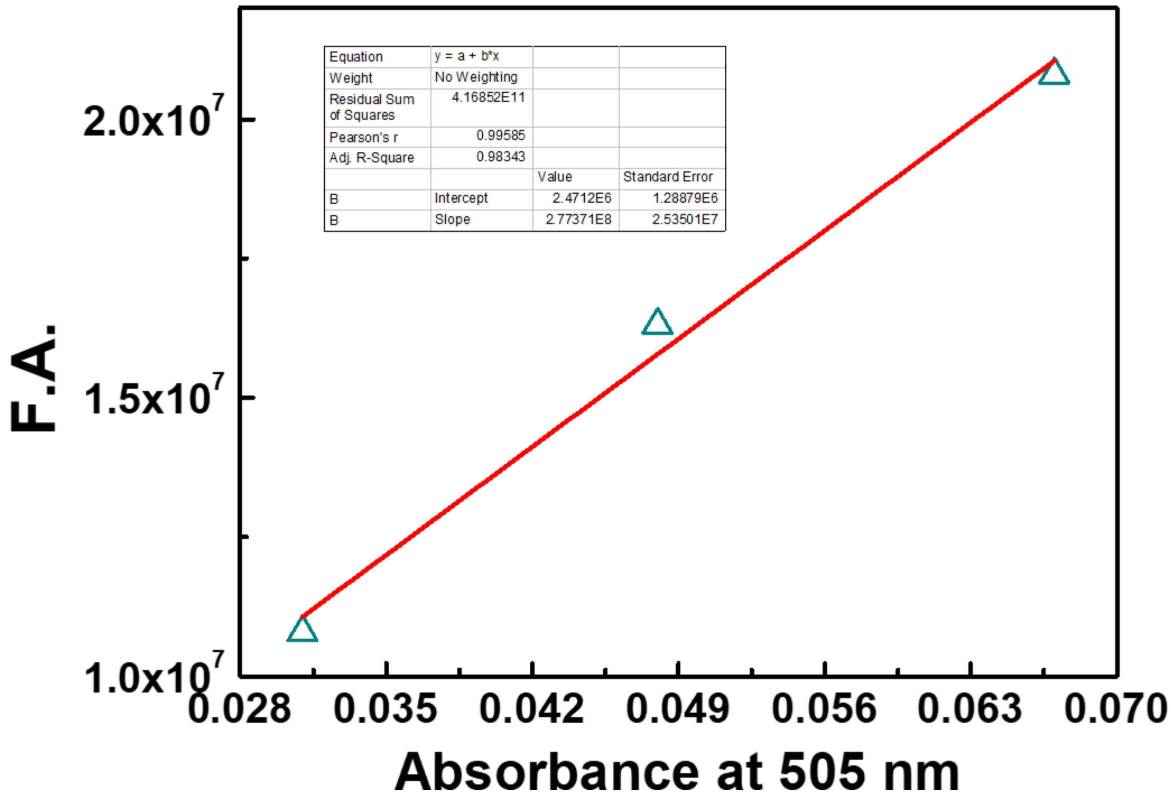

Fig. S15. Gradient plot for Rh6G

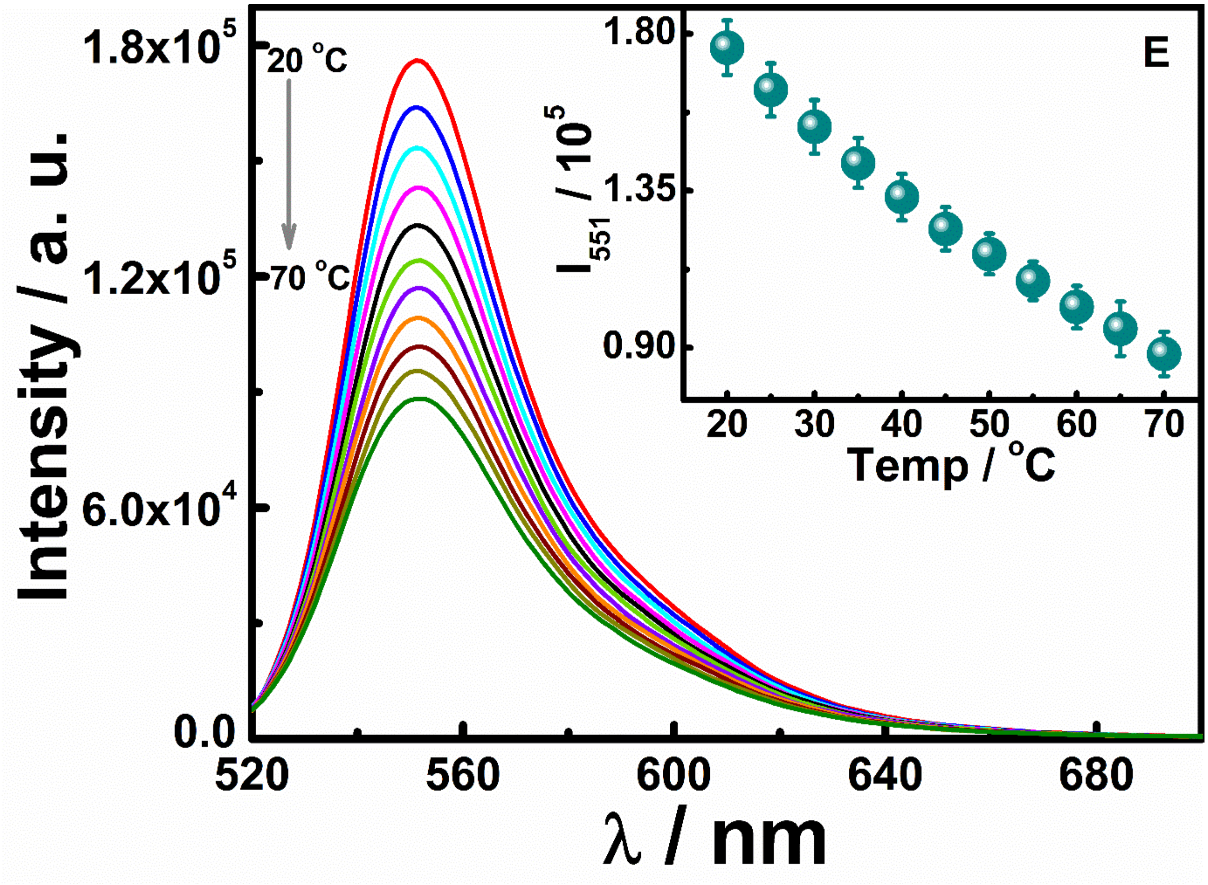

Fig. S16. Temperature dependent study of CND in 10% ethanolic solution.

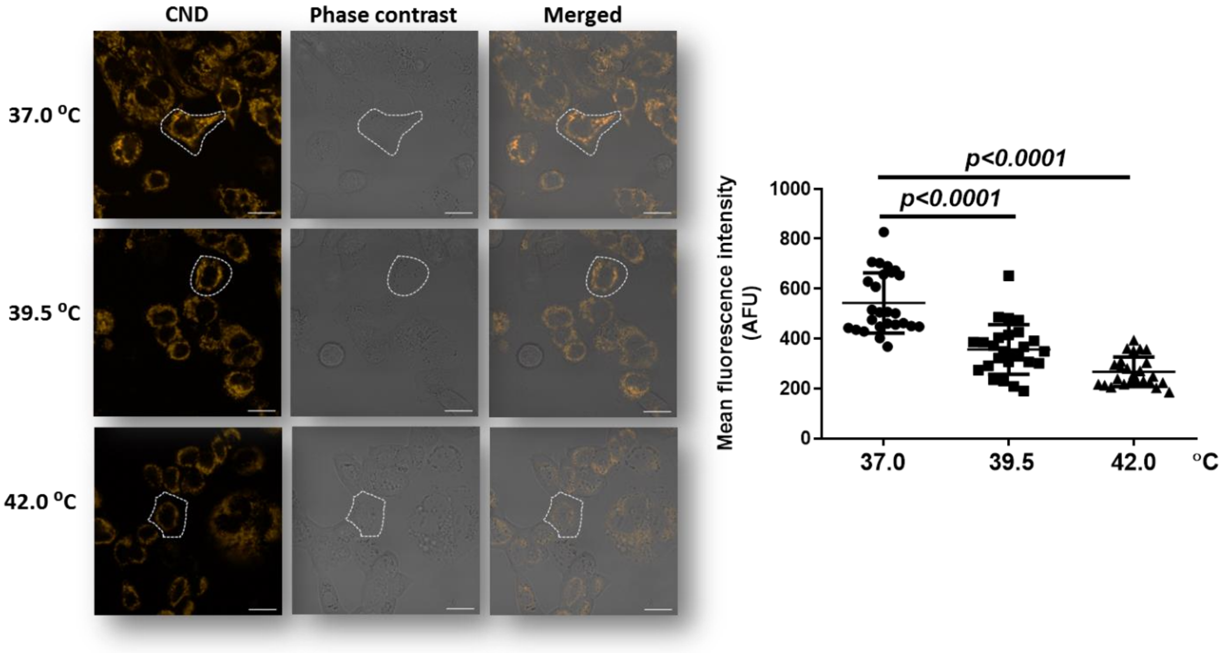

Fig. S17. Microscopic images (fluorescence and bright field) of MCF-7 cells, incubated with CND (5% vol./vol.) for 30 min and kept under different temperature (37- 42 °C) for another 30 min. Quantification of fluorescence of cellular CND, in this experiment, is shown in adjacent plot.

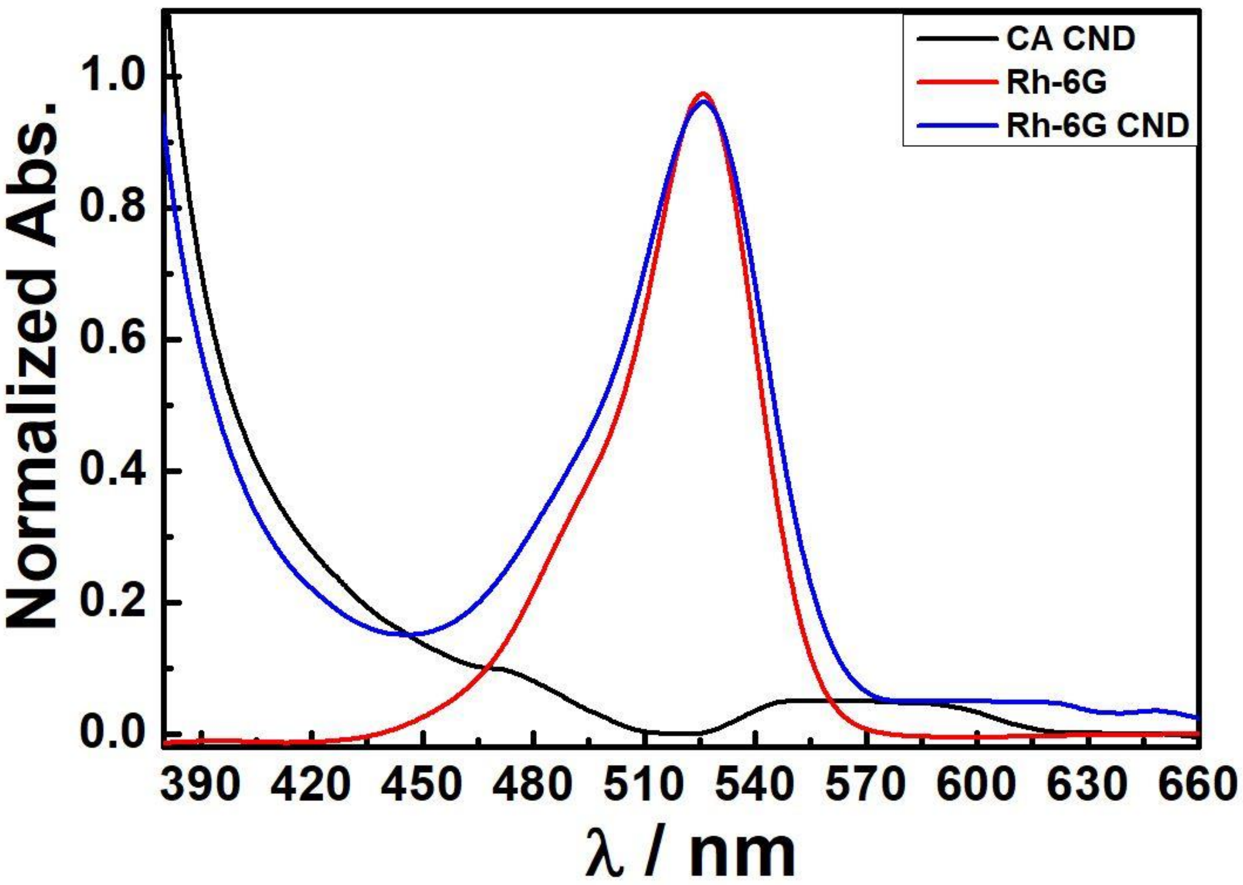

Fig. S18. Normalized UV-VIS spectrum of Citric Acid CND, Rh6G and CND. These spectra were acquired by spectrophotometer.

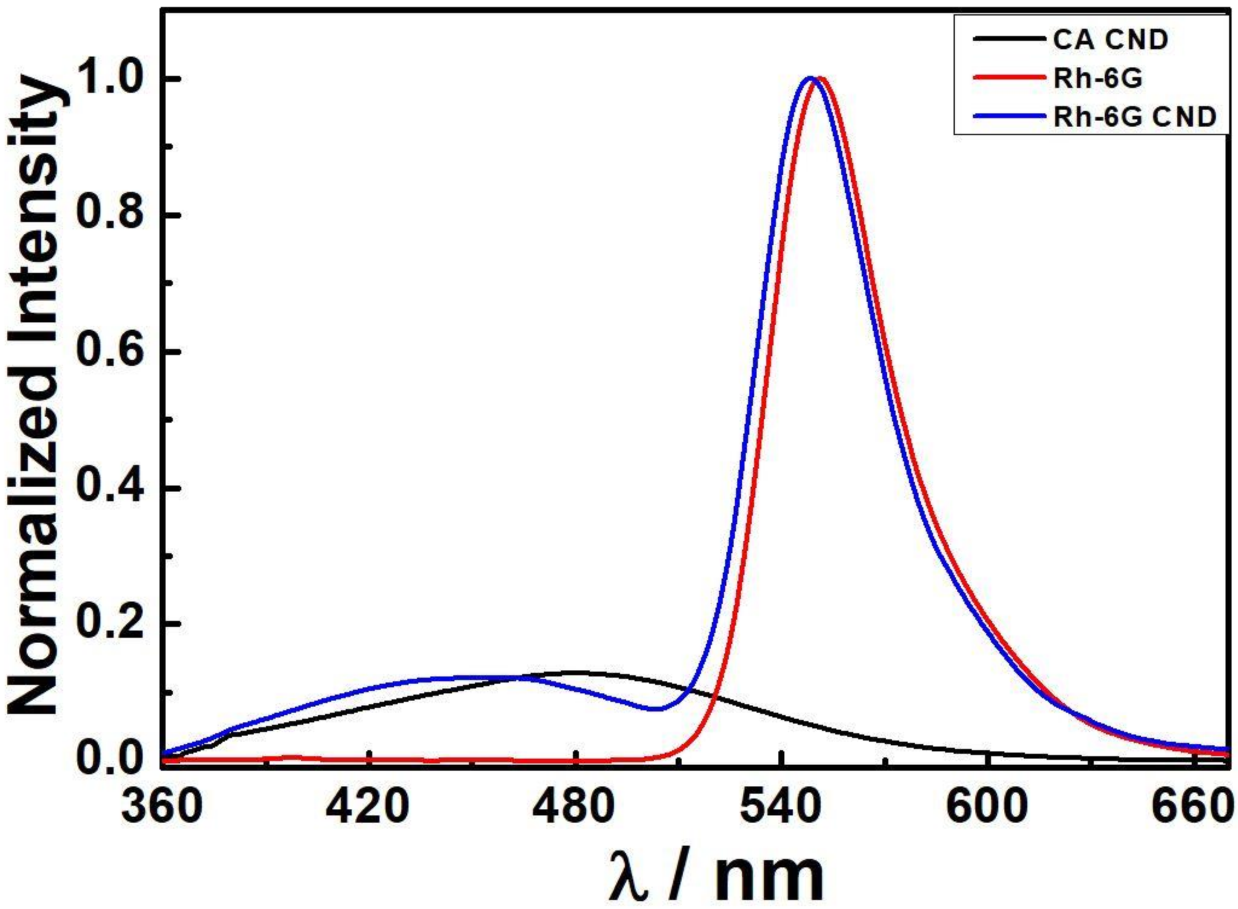

Fig. S19. Normalized Emission spectrum of Citric Acid CND, Rh6G and CND. These spectra were acquired by fluorescence spectrophotometer (λ_{ex} = 350 nm).

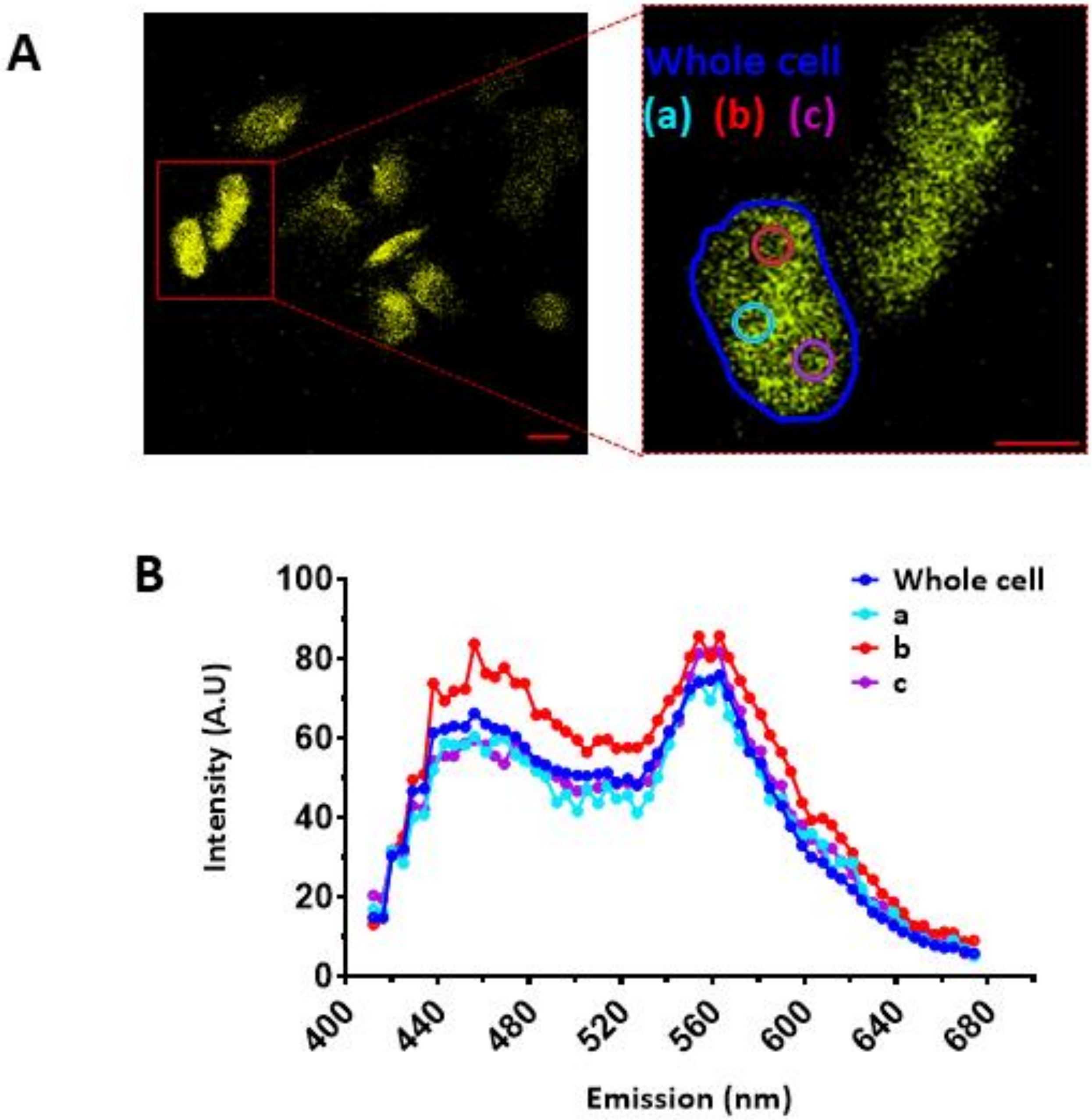
A
Whole cell
(a) (b) (c)
B
Whole cell
a
b
c
Intensity (A.U)
0
20
40
60
80
100
400
440
480
520
560
600
640
680
Emission (nm)

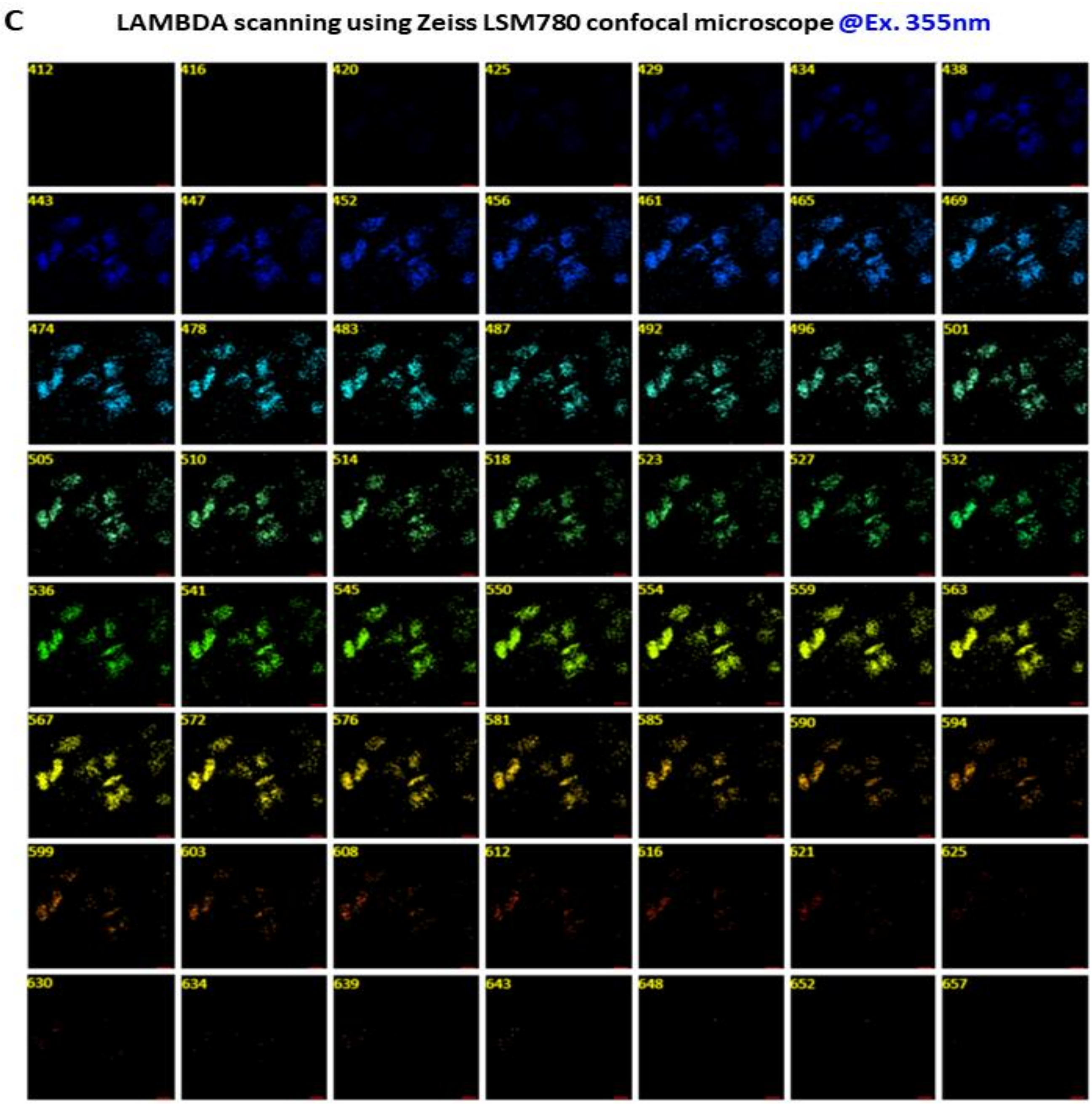

Fig S20. MCF-7 cells were incubated with CND (5% vol./vol.) for 60 min. (A, B) Normalized fluorescence emission spectra of intracellular CND at three random intracellular locations as well as whole cells were acquired by using lamba scanning mode in 780 LSM confocal microscope (l_{ex} = 355 nm; l_{em} = 412-657 nm at the interval of 5 nm). (C) Images taken at 5 nm interval from 412 nm to 657 nm with 355 nm laser excitation were shown

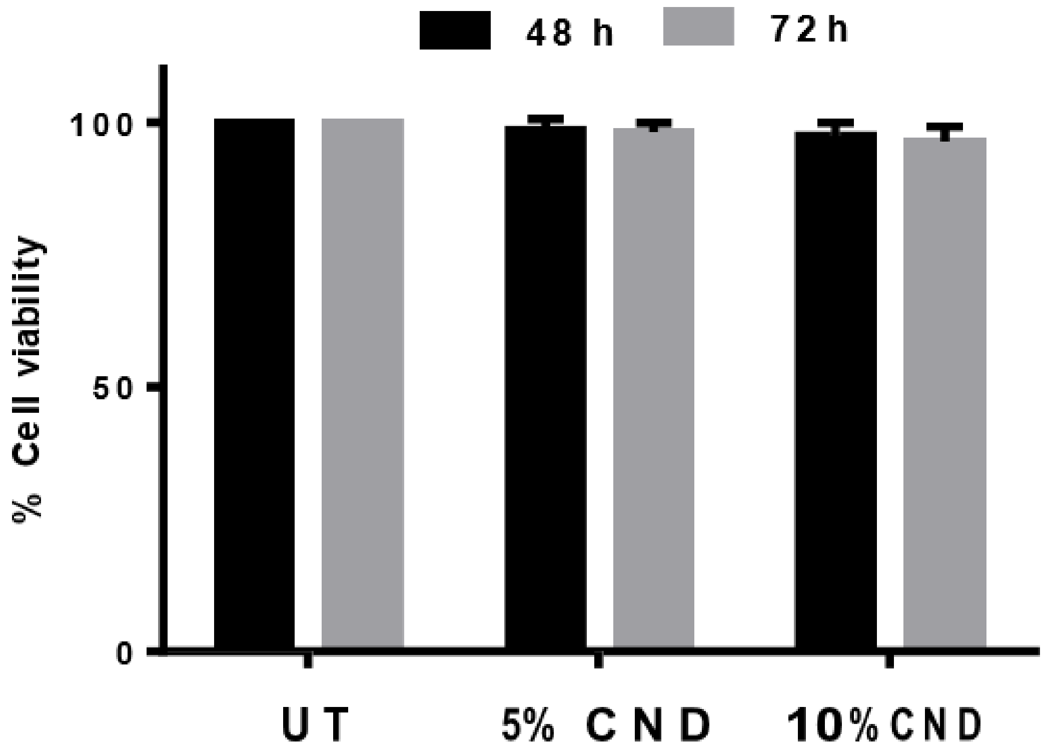

Fig. S21. The effect of CND on cell viability. MCF-7 cells were treated with different concentration of CND for 48-72 h and cell viability was assessed by MTT assay.

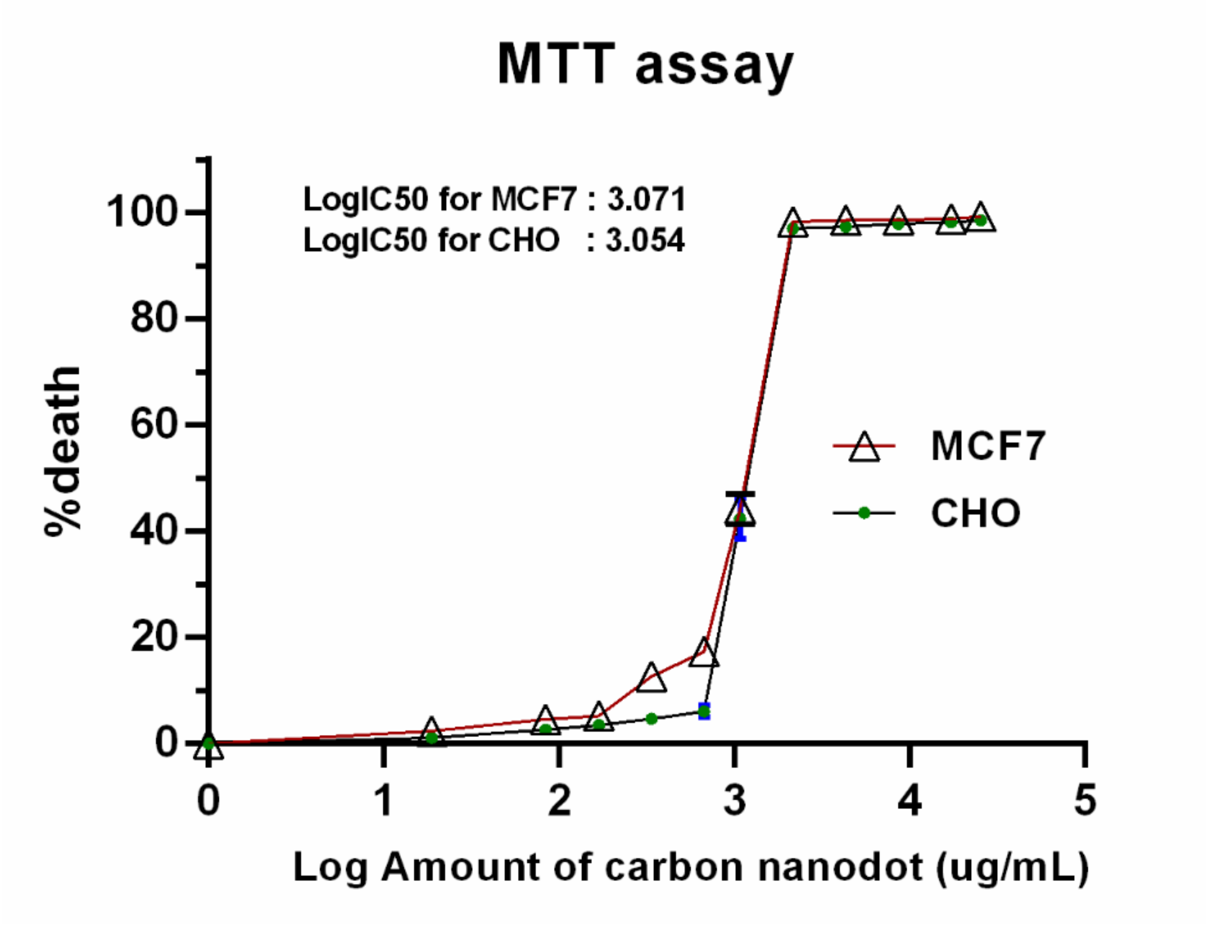

Fig. S22. IC50 values for MCF-7 and CHO cells.

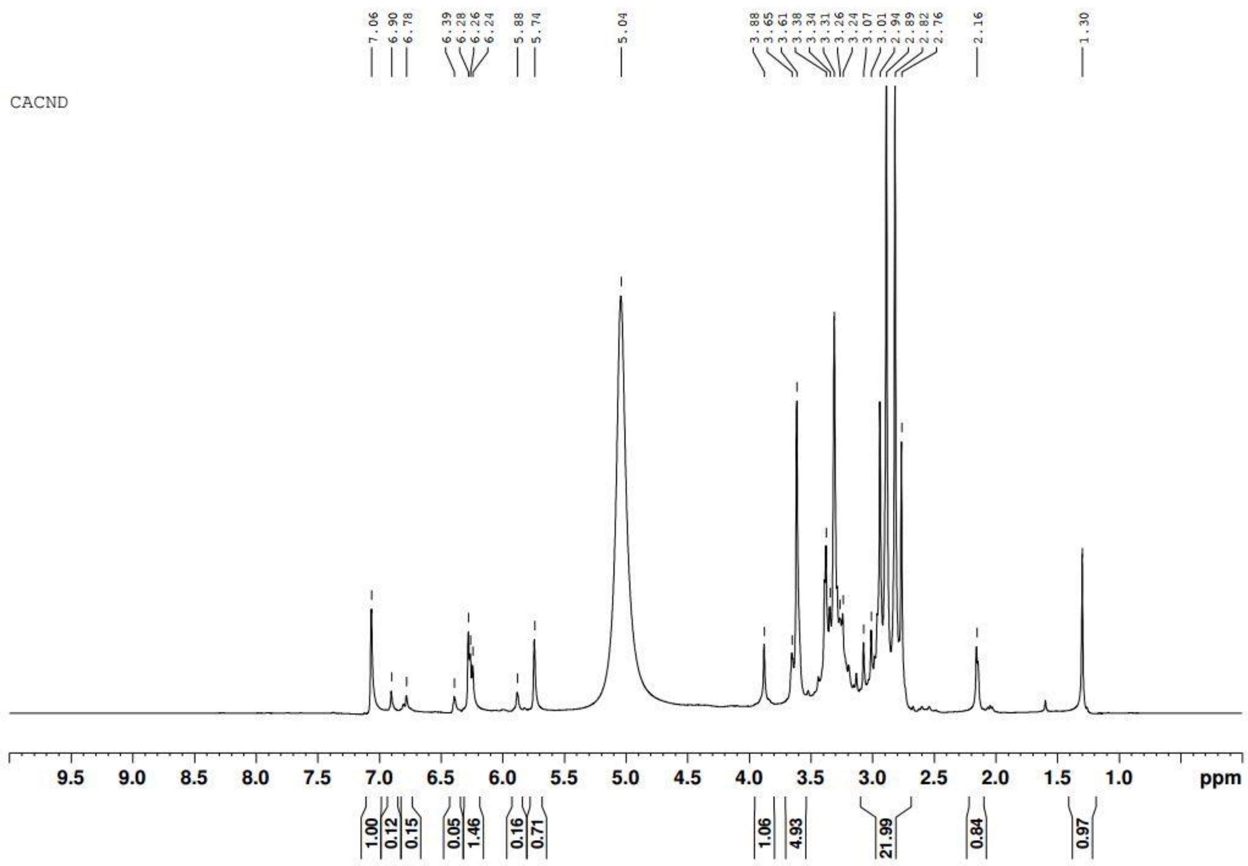

Fig. S23. ^{1}H NMR of Citric acid CND

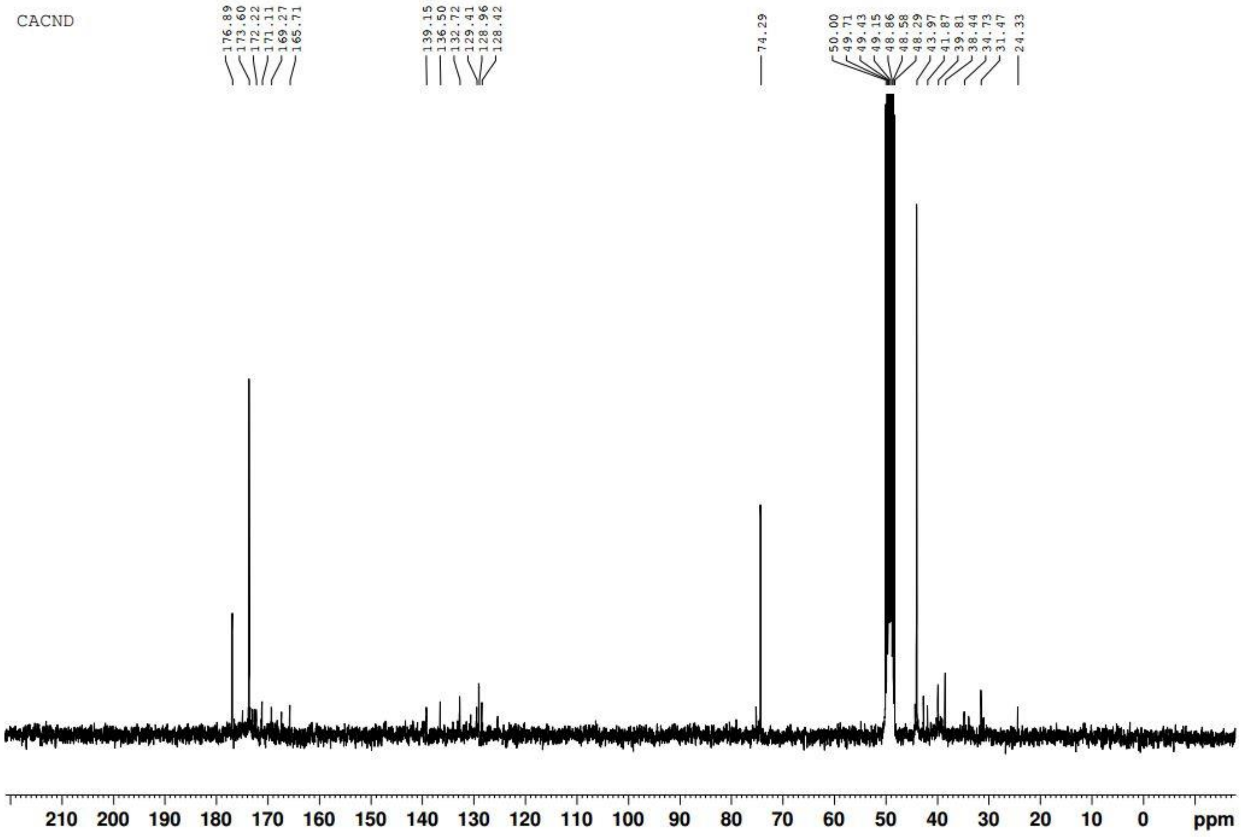

Fig. S24. ^{13}C NMR of Citric acid CND

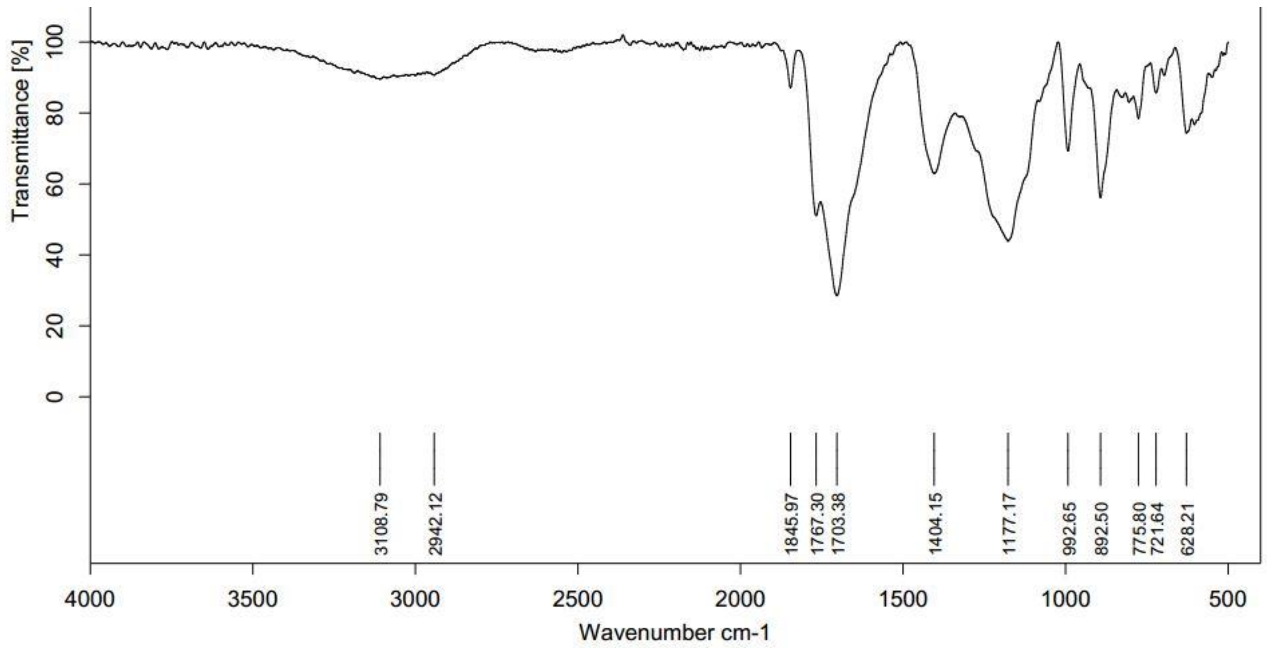

Fig. S25. FT-IR spectrum of Citric Acid CND.

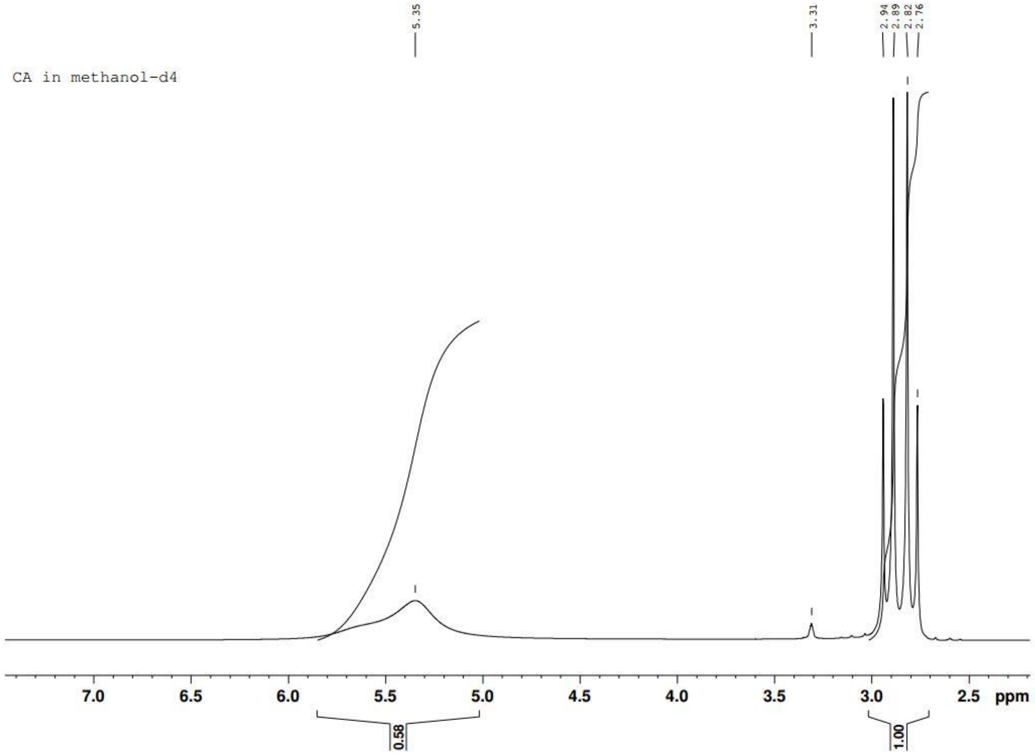

Fig. S26. ^{1}H NMR of Citric acid.

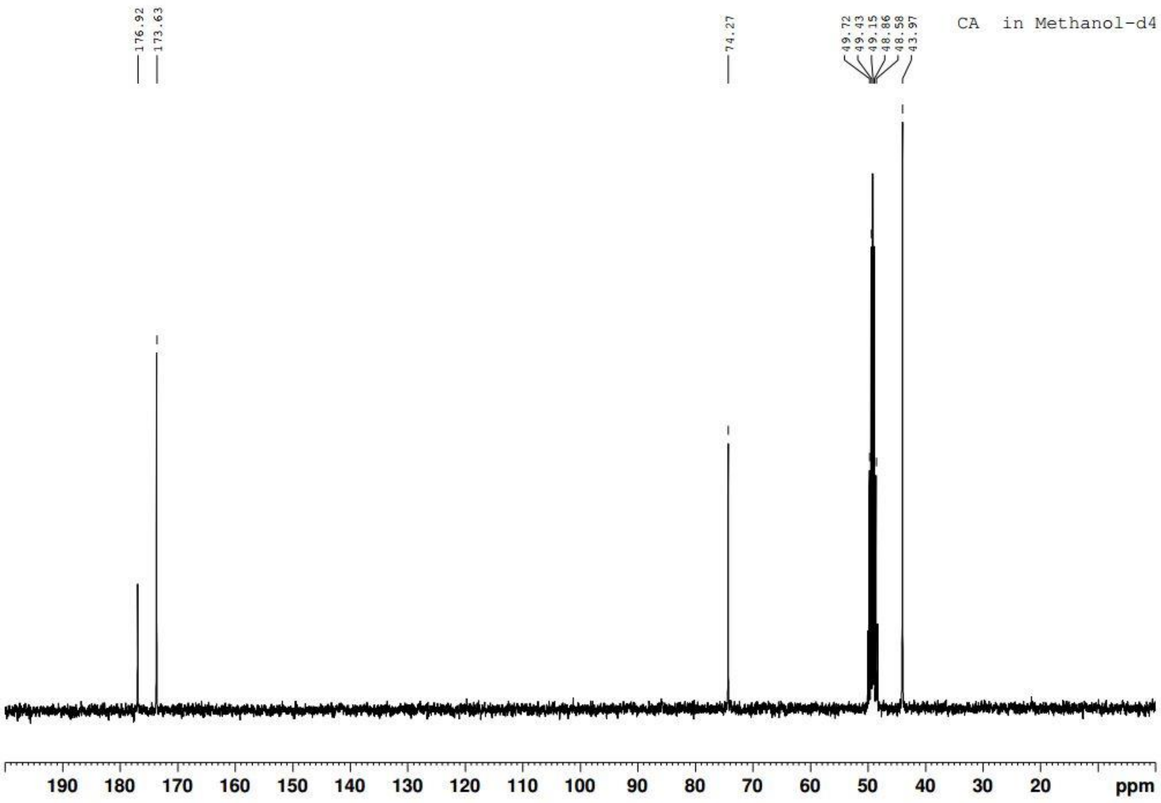

Fig. S27. ^{13}C NMR of Citric acid

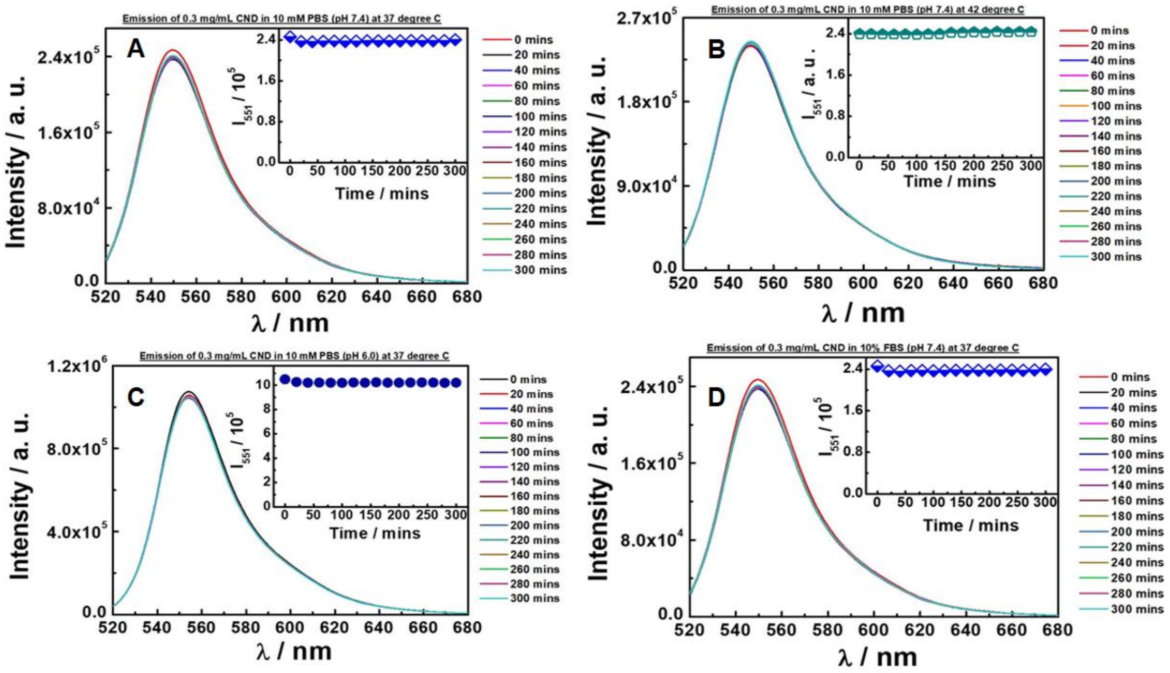

Fig. S28. Stability studies using emission intensity of CND **A)** PL intensity of CND in 10 mM PBS at pH 7.4 at 37 ºC **B)** PL intensity of CND in 10 mM PBS at pH 7.4 at 42 ºC **C)** PL intensity of CND in 10 mM PBS at pH 6.0 at 37 ºC **D)** PL intensity of CND in 10% Fetal Bovine Serum (FBS) at pH 7.4 at 37 ºC.

Table S1. Crystal data ans structure refinement data of Rh6G hydrazide

CCDC number	2051200
Empirical formula	$C_{26}H_{28}N_4O_2$
Formula weight	428.52
Temperature [K]	298(2)
Crystal system	monoclinic
Space group (number)	$P\ 2_1/c$ (14)
a [Å]	17.1920(2)
b [Å]	14.9169(2)
c [Å]	8.83650(10)
α [Å]	90
β [Å]	99.1830(10)
γ [Å]	90
Volume [$Å^3$]	2237.09(5)
Z	4
ρ_{calc} [g/cm^3]	1.272
μ [mm^{-1}]	0.654
$F(000)$	912
Crystal size [mm^3]	0.4 ×0.4 ×0.2
Crystal color	colorless
Crystal shape	plate
Radiation	Cu K_α (λ=1.54184 Å)
2Θ range [°]	5.21 to 154.50 (0.79 Å)
Index ranges	$-21 \le h \le 19$<br>$-11 \le k \le 18$<br>$-10 \le l \le 11$
Reflections collected	16987
Independent reflections	4501<br>R_{int} = 0.0219<br>R_{sigma} = 0.0168
Completeness to θ = 67.684°	99.5 %
Data / Restraints / Parameters	4501/0/300
Goodness-of-fit on F^2	1.040
Final R indexes [$I \ge 2\sigma(I)$]	R_1 = 0.0446<br>wR_2 = 0.1315
Final R indexes [all data]	R_1 = 0.0473<br>wR_2 = 0.1340
Largest peak/hole [$eÅ^3$]	0.37/-0.32
Extinction coefficient	0.0024(4)

Table S2. Bond lengths [Å] and angles [°] of Rh6G hydrazide.

C1–O1	1.3770(15)	O1–C1–C2	123.13(11)
C1–C2	1.3797(17)	O1–C1–C13	114.81(11)
C1–C13	1.3912(18)	C2–C1–C13	122.05(12)
C2–C10	1.3984(17)	C1–C2–C10	116.63(11)
C2–C3	1.5160(17)	C1–C2–C3	122.37(11)
C3–N3	1.4797(16)	C10–C2–C3	121.00(11)
C3–C4	1.5175(17)	N3–C3–C2	110.94(10)
C3–C20	1.5239(18)	N3–C3–C4	111.63(10)
C4–C5	1.3800(18)	C2–C3–C4	109.64(10)
C4–C9	1.4006(18)	N3–C3–C20	99.53(10)
C5–O1	1.3758(16)	C2–C3–C20	112.18(10)
C5–C6	1.3894(18)	C4–C3–C20	112.63(10)
C6–C7	1.389(2)	C5–C4–C9	116.69(12)
C7–N1	1.3802(18)	C5–C4–C3	121.92(11)
C7–C8	1.417(2)	C9–C4–C3	121.39(11)
C8–C9	1.3803(19)	O1–C5–C4	123.56(11)
C8–C17	1.505(2)	O1–C5–C6	114.31(12)
C10–C11	1.3790(18)	C4–C5–C6	122.12(12)
C11–C12	1.4132(18)	C5–C6–C7	120.19(13)
C11–C14	1.5062(18)	N1–C7–C6	120.84(14)
C12–C13	1.3875(18)	N1–C7–C8	119.78(13)
C12–N2	1.3876(17)	C6–C7–C8	119.37(12)
C15–N2	1.4477(19)	C9–C8–C7	118.13(13)
C15–C16	1.513(2)	C9–C8–C17	121.34(14)
C18–N1	1.454(2)	C7–C8–C17	120.53(13)
C18–C19	1.491(3)	C8–C9–C4	123.49(13)
C20–C26	1.378(2)	C11–C10–C2	123.53(12)
C20–C21	1.3847(18)	C10–C11–C12	118.22(11)
C21–C23	1.3959(19)	C10–C11–C14	121.41(12)
C21–C22	1.477(2)	C12–C11–C14	120.36(12)
C22–O2	1.2302(17)	C13–C12–N2	122.08(12)
C22–N3	1.3577(17)	C13–C12–C11	119.36(11)
C23–C24	1.375(3)	N2–C12–C11	118.55(12)
C24–C25	1.381(3)	C12–C13–C1	120.18(12)
C25–C26	1.392(2)	N2–C15–C16	113.46(13)
N3–N4	1.4020(16)	N1–C18–C19	110.41(18)
		C26–C20–C21	120.92(13)
		C26–C20–C3	128.62(12)
		C21–C20–C3	110.43(11)
		C20–C21–C23	121.22(14)
		C20–C21–C22	108.93(11)
		C23–C21–C22	129.82(13)
		O2–C22–N3	124.99(14)
		O2–C22–C21	129.46(13)
		N3–C22–C21	105.55(11)
		C24–C23–C21	117.76(15)
		C23–C24–C25	120.90(15)
		C24–C25–C26	121.61(16)
		C20–C26–C25	117.57(15)
		C7–N1–C18	122.73(14)
		C12–N2–C15	121.27(12)
		C22–N3–N4	124.31(11)

C22–N3–C3	115.18(11)
N4–N3–C3	119.27(10)
C5–O1–C1	117.78(10)

Table S3. Torsion angles [°] for Rh6G hydrazide

O1–C1–C2–C10	-179.72(12)
C13–C1–C2–C10	1.07(19)
O1–C1–C2–C3	0.5(2)
C13–C1–C2–C3	-178.75(12)
C1–C2–C3–N3	-133.97(12)
C10–C2–C3–N3	46.22(16)
C1–C2–C3–C4	-10.22(17)
C10–C2–C3–C4	169.97(11)
C1–C2–C3–C20	115.70(13)
C10–C2–C3–C20	-64.11(15)
N3–C3–C4–C5	134.55(13)
C2–C3–C4–C5	11.21(17)
C20–C3–C4–C5	-114.46(14)
N3–C3–C4–C9	-46.46(16)
C2–C3–C4–C9	-169.80(12)
C20–C3–C4–C9	64.53(16)
C9–C4–C5–O1	178.37(12)
C3–C4–C5–O1	-2.6(2)
C9–C4–C5–C6	-0.6(2)
C3–C4–C5–C6	178.47(13)
O1–C5–C6–C7	-178.01(13)
C4–C5–C6–C7	1.0(2)
C5–C6–C7–N1	178.89(14)
C5–C6–C7–C8	-0.8(2)
N1–C7–C8–C9	-179.51(14)
C6–C7–C8–C9	0.2(2)
N1–C7–C8–C17	0.4(2)
C6–C7–C8–C17	-179.85(15)
C7–C8–C9–C4	0.2(2)
C17–C8–C9–C4	-179.70(14)
C5–C4–C9–C8	-0.1(2)
C3–C4–C9–C8	-179.11(13)
C1–C2–C10–C11	-0.4(2)
C3–C2–C10–C11	179.46(12)
C2–C10–C11–C12	-0.9(2)
C2–C10–C11–C14	178.07(12)
C10–C11–C12–C13	1.48(19)
C14–C11–C12–C13	-177.50(12)
C10–C11–C12–N2	179.84(12)
C14–C11–C12–N2	0.86(19)
N2–C12–C13–C1	-179.13(12)
C11–C12–C13–C1	-0.8(2)
O1–C1–C13–C12	-179.76(12)
C2–C1–C13–C12	-0.5(2)
N3–C3–C20–C26	179.56(13)

C2–C3–C20–C26	-63.07(17)
C4–C3–C20–C26	61.21(17)
N3–C3–C20–C21	-2.16(13)
C2–C3–C20–C21	115.21(12)
C4–C3–C20–C21	-120.51(12)
C26–C20–C21–C23	-1.3(2)
C3–C20–C21–C23	-179.71(12)
C26–C20–C21–C22	176.93(12)
C3–C20–C21–C22	-1.51(14)
C20–C21–C22–O2	-174.63(14)
C23–C21–C22–O2	3.4(2)
C20–C21–C22–N3	4.91(14)
C23–C21–C22–N3	-177.10(13)
C20–C21–C23–C24	0.3(2)
C22–C21–C23–C24	-177.52(14)
C21–C23–C24–C25	1.1(2)
C23–C24–C25–C26	-1.4(3)
C21–C20–C26–C25	0.9(2)
C3–C20–C26–C25	179.05(13)
C24–C25–C26–C20	0.4(2)
C6–C7–N1–C18	-0.2(3)
C8–C7–N1–C18	179.52(17)
C19–C18–N1–C7	-178.3(2)
C13–C12–N2–C15	-14.6(2)
C11–C12–N2–C15	167.07(12)
C16–C15–N2–C12	-66.57(19)
O2–C22–N3–N4	5.7(2)
C21–C22–N3–N4	-173.89(12)
O2–C22–N3–C3	172.82(13)
C21–C22–N3–C3	-6.75(14)
C2–C3–N3–C22	-112.66(12)
C4–C3–N3–C22	124.74(12)
C20–C3–N3–C22	5.65(13)
C2–C3–N3–N4	55.19(15)
C4–C3–N3–N4	-67.42(14)
C20–C3–N3–N4	173.49(11)
C4–C5–O1–C1	-8.4(2)
C6–C5–O1–C1	170.58(12)
C2–C1–O1–C5	9.5(2)
C13–C1–O1–C5	-171.23(12)

Table. S4. FT-IR spectrum analysis of CND (Fig. S8) with functional groups and corresponding wavenumber.

**Functional Groups**	**Wavenumber**
N-H stretch	3494.5 cm^{-1}
C-H stretch	2988.8 cm^{-1}
O-H stretch	2533.4 cm^{-1}
C=N and C=O stretch (Amide Group)	1701.6 cm^{-1}, 1633.6 cm^{-1}
C-N stretch (for Aromatic Amine)	1400.5 cm^{-1}
C-N stretch	1186.7 cm^{-1}
C-O stretch	973.5 cm^{-1}

Elemental Analysis

Table S5. Elemental composition of CND

	Carbon	Hydrogen	Nitrogen	Oxygen
CND	43%	12%	0.1%	44.9%
